# Photo-PISA Driven *In Situ* Encapsulation of Nanocluster-Based Sensors within Stimuli-Responsive Polymersomes for AND Logic Gate Sensing

**DOI:** 10.1021/acssensors.5c02685

**Published:** 2025-11-02

**Authors:** Kaili Chen, Colleen N. Loynachan, Chalaisorn Thanapongpibul, Junni Zhang, Liyun Ma, Jonathan Yeow, Adrian Najer, Molly M. Stevens

**Affiliations:** † Department of Materials, Department of Bioengineering, 4615 Institute of Biomedical Engineering, Imperial College London, SW7 2AZ London, U.K.; ‡ Department of Physiology, Anatomy and Genetics, Department of Engineering Science, 6396 Kavli Institute for Nanoscience Discovery, University of Oxford, OX1 3QU Oxford, U.K.

**Keywords:** photoinitiated polymerization-induced self-assembly, catalytic gold nanocluster, polymersome, enzyme-responsive, pH-responsive

## Abstract

Colorimetric sensing is a widely utilized analytical technique due to its simplicity, accessibility, rapid response, and broad applicability in medical diagnostics. However, improving the sensitivity and specificity of these assays remains a critical challenge in complex disease states, especially when sensing endogenous enzymes as biomarkers. In this study, we have developed a hierarchical AND logic gate dual-sensing platform that integrates peptide-templated, catalytically active gold nanoclusters (AuNCs), acting as nanozymes tethered to a carrier protein (AuNC-protein complex nanosensor), and loads them within pH-responsive polymersomes synthesized via *in situ* photoinitiated polymerization-induced self-assembly (photo-PISA). Under physiological conditions, the AuNC-protein complex is stably encapsulated within the enzyme-impermeable polymersome but becomes released under acidic conditions. In the presence of a target enzyme, the AuNCs can then be cleaved from the supramolecular protein complex, separated, and quantified by a colorimetric readout, yielding a positive signal only when the sensor encounters both an acidic environment and the target enzyme. This AND logic gate design minimizes background signals and enhances specificity, making it particularly suitable for complex biological environments. We envision future use of this system for dual-responsive *in vivo* sensing of overexpressed enzymes in acidic tumor or inflammatory microenvironments, with a simple colorimetric urinary readout.

## Graphical abstract

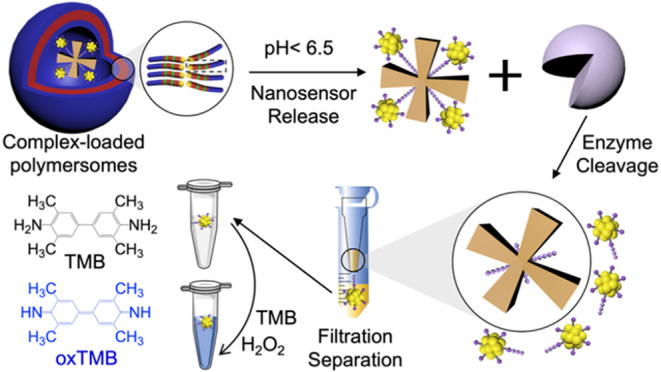

In medical diagnostics and disease monitoring, simple, low-cost, and sensitive diagnostics are crucial for real-time analysis and early diagnosis. While traditional chromatographic and spectrometric techniques offer high precision and accuracy, they often require complex instrumentation, which limits their accessibility and applicability for on-site disease detection and monitoring.^1,2^ Hence, there is growing demand for developing simple, efficient, and visually readable diagnostic techniques that do not rely on complex equipment but are affordable and easy to operate. Colorimetric methods, which produce visible color changes, have received attention for their simplicity, low cost, and suitability for rapid, on-site testing.^3–5^ However, enhancing the specificity and sensitivity of colorimetric methods remains a significant challenge, requiring further exploration of alternative approaches such as AND logic gate sensing mechanisms, in which an output signal is produced only when two distinct input conditions are present,^6,7^ thereby improving detection reproducibility and specificity by reducing false positives from single-stimulus activation.

Catalytic gold nanoclusters (AuNCs) have emerged as a versatile nanomaterial due to their exceptional optical properties and catalytic potential, showing significant promise for applications in colorimetric sensing.^8–11^ Extensive studies have demonstrated their excellent biocompatibility and unique size-dependent properties, highlighting their strong potential for *in vivo* applications, including biosensing and drug delivery.^12–15^ AuNCs exhibit unique electronic structures that enable them to function as effective catalysts (nanozymes) in colorimetric reactions. This allows for rapid and visible color changes in the presence of target biomolecules or substances.^16,17^ A common colorimetric reaction employs 3,3′,5,5′-tetramethylbenzidine (TMB) in the presence of hydrogen peroxide (H_2_O_2_), where AuNCs catalyze the oxidation of TMB (a color change from colorless to blue), significantly improving detection sensitivity by the naked eye.^9,10,18,19^ Moreover, AuNCs offer remarkable versatility for functionalization, either via templating or conjugation with peptides, facilitating the design of complex enzyme sensor systems.^20,21^ Their ultrasmall size (typically < 5 nm) allows easy separation from larger carriers, including *in vivo*, allowing clearance through the kidneys and direct colorimetric readout in the urine.^10^

On a larger length scale (typically 20–500 nm), polymeric nanoparticles (NPs) offer compartments for encapsulation of various sensing units, enabling multiplexed detection and endowing stimuli-responsive behavior.^22–24^ Recent advances in polymeric NPs have further expanded this toolbox, allowing their scalable synthesis with controllable morphologies (e.g., polymersomes) and tunable responsiveness.^25–27^ For instance, polymerization-induced self-assembly (PISA)-derived polymersomes have been engineered for glucose sensing^28^ and drug delivery,^29^ demonstrating their potential for biomedical applications. One possibility is to sense acidic environments with pH-responsive polymeric NPs, like polymersomes, offering innovative strategies for the design of advanced biosensors.^30,31^ pH-sensitive polymersomes can be engineered to undergo structural transformations, such as morphological changes with an accompanied release of encapsulated contents under acidic conditions.^32–36^ This property can be leveraged for therapeutic and biosensing applications that target acidic environments such as tumors or sites of inflammation.^37–40^ Dual-responsive polymeric NPs have been designed to require two stimuli for controlled release of cargo, including sensing probes.^31^ These advances highlight the versatility of polymeric NPs as powerful platforms for next-generation biomedical sensing applications.

Dual-responsive and logic-gated biosensors have emerged as an attractive strategy to improve specificity in complex biological environments.^6,7,41^ Nanoparticle-based strategies for harnessing multiple stimuli in sensing include lipid-based NPs^42–44^ and polymeric NPs^31,45,46^ that respond to two or more stimuli, incorporating AND- or OR-gate recognition.

In this study, we have developed an advanced AND logic gate sensing platform by combining the catalytic properties of AuNCs with the versatility of enzyme-responsive peptide-linked complexes and the environmental adaptability of pH-sensitive polymersomes (Figure 1). This platform provides an AND logic gate that generates a colorimetric signal only in the presence of an acidic pH and the presence of specific enzymes. Initially, we optimized the peptide-to-AuNC ratio to enhance the catalytic performance of the enzyme-responsive nanosensors. These peptide-templated AuNC nanosensors were subsequently encapsulated within pH-responsive polymersomes via photoinitiated-PISA (photo-PISA) to form the AND gate sensing platform. This sensing platform generated a positive signal from released catalytic AuNCs only in environments that met both criteria, i.e., pH < 6.5 and the presence of target enzyme. This strategy could reduce the risk of false positives of single-condition scenarios as the outer polymersome is impermeable to enzymes at physiological pH. Our AND logic gate sensor allowed sensitive and specific detection of two specific biomarkers with potential applications in diagnostics and disease monitoring. We envision future use of this AND gate sensing system for *in vivo* diagnostic applications, providing a signal only after pH- and enzyme-mediated disassembly and AuNC liberation, which could then be detected in urine via a catalytic colorimetric reaction.

**Figure 1:**
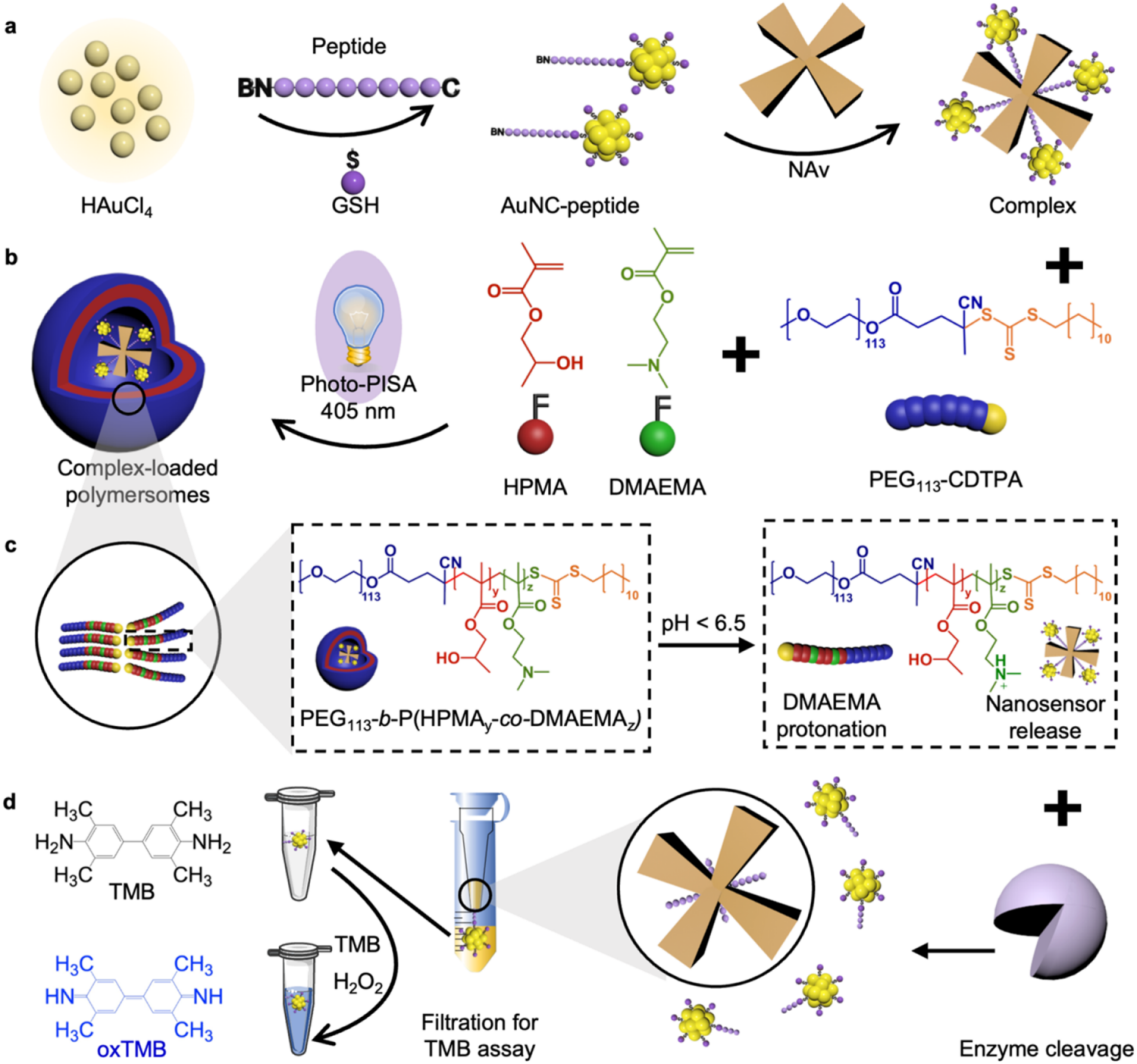
Design of an AND-gated sensing platform including enzyme-responsive nanosensors encapsulated in pH-responsive polymersomes. (a) Synthesis of peptide-templated AuNCs and assembly of the enzyme-responsive nanosensor. Gold­(III) was reduced to gold (0) by GSH and cysteine-terminated peptides, resulting in the self-assembly of peptide-templated AuNCs with a biotin at the opposite end. They were subsequently conjugated to NAv via the biotin ends, forming an enzyme-responsive nanosensor. (b) Photo-PISA schematic showing the encapsulation of enzyme-responsive nanosensors. Polymersomes were formed through photo-PISA with 405 nm light irradiation, encapsulating the enzyme-responsive nanosensors. (c) Spherical bilayer structure of the amphiphilic block copolymer after photo-PISA synthesis. pH responsiveness arises from the protonation of DMAEMA units in the polymersome membrane under acidic conditions, causing disassembly of polymersomes due to the formation of soluble copolymers. (d) Enzyme-mediated cleavage of nanosensors released from polymersomes, enabling the separation of the released AuNCs for the TMB assay, in which AuNCs catalyze the oxidation of TMB to yield a blue color.

## Results and Discussion

### Optimization of Enzyme-Responsive Nanosensor Complexes

The AND-gated sensor design involves hierarchical encapsulation of a small nanosensor complex in pH-responsive polymersomes. Catalytically active AuNCs were tethered to a carrier protein via enzyme-responsive linkages,^10^ which were then encapsulated in pH-responsive polymersomes^36^ via photo-PISA (Figure 1). First, the AuNC-protein complexes were optimized. We synthesized thrombin- and matrix metallopeptidase 9 (MMP-9)-specific peptides through solid-phase peptide synthesis (SPPS) following our previous work (Table S1 and Figure S1).^10^ These peptides are terminated with a gold-binding cysteine on one end and a biotin on the other. AuNCs were synthesized in the presence of a specific ratio of these peptides to glutathione (GSH), followed by purification and tethering to a protein carrier neutravidin (NAv) to achieve the enzyme-responsive nanosensor (Figure 2a).

**Figure 2:**
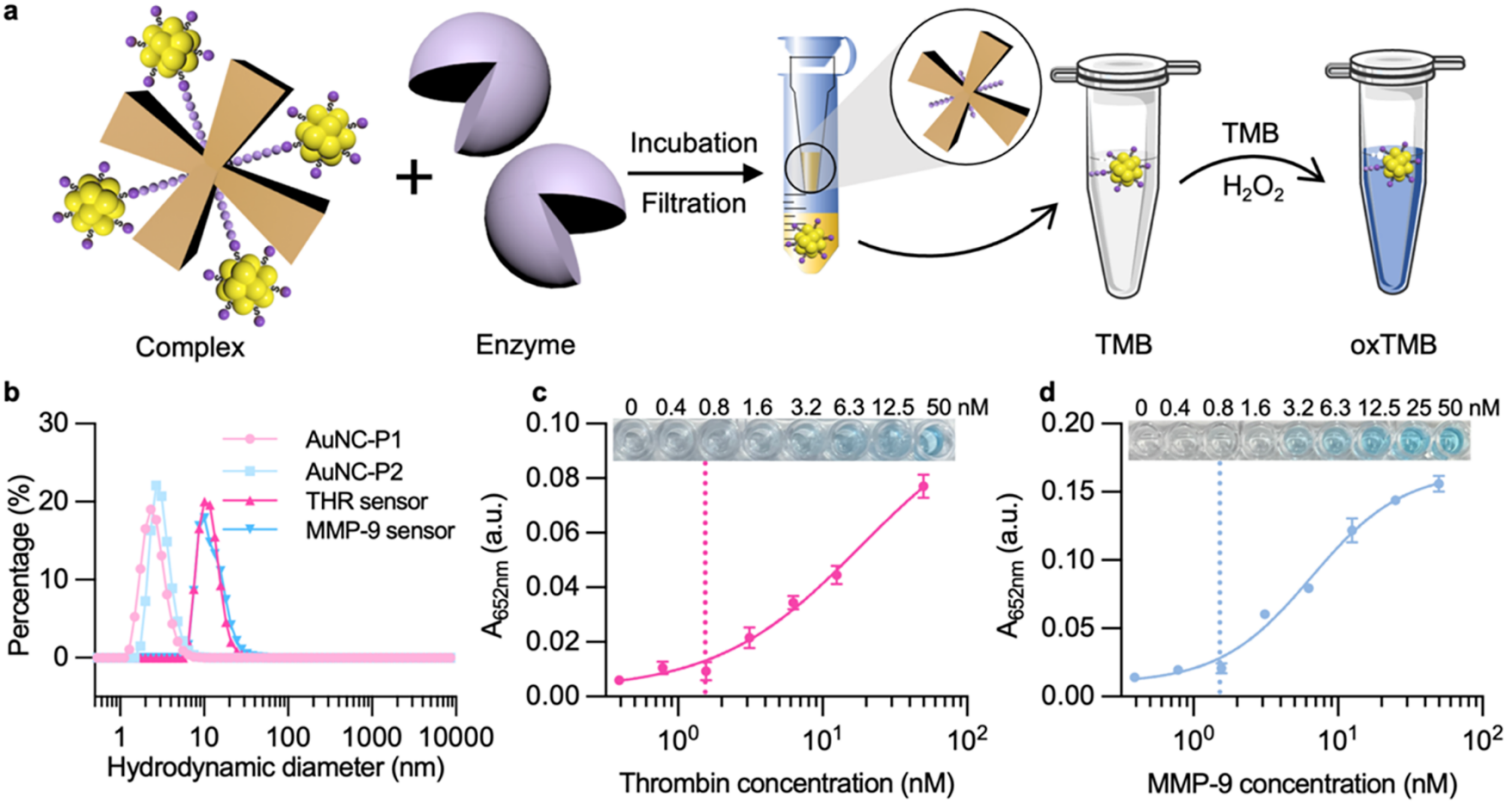
Characterization and *in vitro* sensing performance of thrombin- and MMP-9-responsive nanosensor complexes. (a) Schematic of the *in vitro* sensing mechanism of the enzyme-responsive nanosensor. The nanosensors were incubated with the corresponding enzyme, which cleaved the specific peptide, releasing catalytic AuNCs. Amicon filter devices (MWCO 30 kDa) separated intact complexes (in the membrane) and released AuNCs (in the filtrate). The filtrates were collected for TMB assay, which involves AuNC-catalyzed oxidation of TMB in the presence of H_2_O_2_. (b) Dynamic light scattering (DLS) of thrombin peptide-templated AuNCs (AuNC-P1) and MMP-9 peptide-templated AuNCs (AuNC-P2), and their corresponding NAv complexes, called THR sensor and MMP-9 sensor, respectively (number distribution from average *n* = 3 technical replicates). (c, d) *In vitro* cleavage performance of THR sensor and MMP-9 sensor when treated with different concentrations of thrombin and MMP-9, respectively (mean value ± standard deviation, *n* = 3 technical replicates). The data were fitted with a four-parameter logistic regression function. The dashed line indicates the limit of detection (LoD) defined as the concentration giving 10% of the maximal absorbance of the fitted curve.

Here, we optimized the peptide density on the AuNC surface to enhance the cleavage efficiency of the nanosensor by the specific enzyme (Figure S2). Through biotin quantification, we confirmed that the higher the percentage of peptide used in the AuNC synthesis, the more peptide was present on the AuNC surface. AuNC yields were similar for all of the thrombin-cleavable peptide (P1) percentages and increased with higher MMP-9-cleavable peptide (P2) ratios, which led to higher signals when performing the catalytic reaction (Figure S2c–f). The percentage of cleavable peptide used in the AuNC synthesis correlated only with a slight size increase (all < 5 nm mean diameter) at constant slightly negative zeta potentials (Figure S3).

The influence of different peptide:GSH ratios on the nanosensor performance was verified by conjugating the library of AuNCs with varying peptide ratios to NAv at constant overall peptide (biotin) concentration. The sizes of the obtained nanosensors were similar at around 10 nm, slightly larger than bare NAv and with a negative zeta potential (Figure S4). TMB oxidation assay revealed that lower peptide density on AuNCs resulted in a higher catalytic activity for the nanosensors, with an optimum at around 2 mol % cleavable peptide and 98 mol % GSH used in the AuNC synthesis (Figure S5a–d). UV–vis absorbance measurements showed that a higher concentration of purified nanosensors was synthesized when using AuNCs with a low percentage of peptide vs GSH. This is likely due to a better conjugation efficiency with NAv when keeping the concentration of peptide constant, consistent with the catalytic activity analysis (Figure S5e,f). NAv conjugation efficiency was found to be highest for the intermediate percentage of about 2–5 mol % cleavable peptides (Table S2).

Next, the enzyme-responsiveness of the nanosensor library was assessed. After incubation with the target enzymes, centrifugal filters (MWCO 30 kDa) were used to separate liberated AuNC from the protein NAv carrier. This step not only enabled a clear readout of catalytic activity but also served as an *in vitro* mimic of kidney clearance. (Figure 2a). While the currently intended use of our sensor system is for *in vivo* sensing, alternative approaches that allow direct signal activation without size separation could be envisioned to permit a one-pot assay for *in vitro* sensing purposes. The signal from the nanosensor system made with AuNCs with a 2 mol % cleavable peptide percentage, which corresponds to 1–2 biotinylated peptides per AuNC (Figure S2a,b), showed the best performance for both enzymes, either by naked-eye readout or absorbance measurements at 652 nm (Figure S6). Therefore, we used nanosensors made with AuNCs containing 2 mol % cleavable peptides. Hydrodynamic diameters of AuNCs with the optimized 2 mol % ratio and their corresponding nanosensors had a mean diameter of about 2 and 10 nm, respectively (Figure 2b), which was further verified with transmission electron microscopy (TEM) (Figure S4e, f). The optimized THR sensor and MMP-9 sensor both exhibited LoD in the range 1–2 nM, determined as 10% of the maximum absorbance signal from fits using a four-parameter logistic eq (Figure 2c,d).

### Preparation, Optimization, and Characterization of pH-Responsive Polymersomes

To realize an AND logic gate sensing platform, a pH-responsive polymersome was designed to serve as a secondary carrier for the nanosensors (Figure 1b). First, 4-cyano-4-[(dodecylsulfanylthiocarbonyl)­sulfanyl] pentanoic acid modified poly­(ethylene glycol) monomethyl ether (PEG_113_-CDTPA) was synthesized and purified according to published work (Figure S7).^47^ Polymersomes were formed through a photo-PISA reaction under illumination with a 405 nm LED array (Figure 3a). PEG_113_-CDTPA was employed as the polymermeic photoinitiator and chain transfer agent, in the presence of 2-hydroxypropyl methacrylate (HPMA) and 2-(dimethylamino)­ethyl methacrylate (DMAEMA) as monomers.^36^ The pH responsiveness is attributed to the presence of DMAEMA, which becomes protonated when pH < 6.5, increasing the hydrophilicity of the hydrophobic polymer block and inducing polymersome disassembly.^48,49^

**Figure 3:**
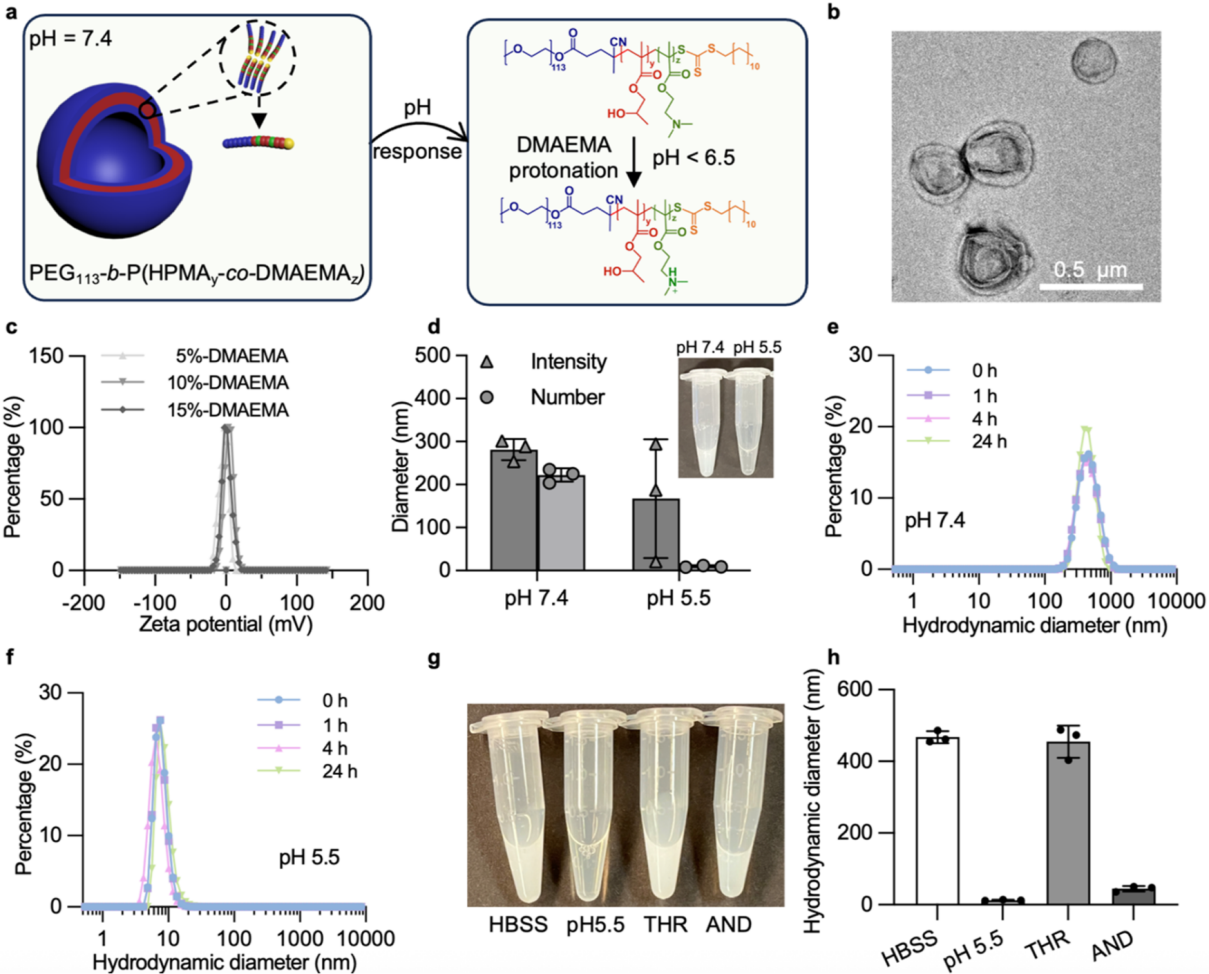
Optimization and characterization of pH-responsive polymersomes. (a) Schematic of the proposed polymersome responsiveness to low pH. (b) TEM image of polymersomes with 10 mol % DMAEMA relative to HPMA (a total of 12 images were taken with similar results). (c) Zeta potential measurements of polymersomes synthesized at different percentages of DMAEMA (mean of *n* = 3 technical replicates). (d) DLS measurements of polymersomes with 10 mol % DMAEMA at pH 7.4 and pH 5.5, showing mean intensity- and number-derived hydrodynamic diameters (insert: photograph of a 10 mol % DMAEMA polymersome suspension at pH 7.4 and pH 5.5). (e, f) Stability testing of polymersomes with 10 mol % DMAEMA by DLS after incubation at 37 °C at (e) pH 7.4 and (f) pH 5.5 for up to 24 h (mean of *n* = 3 technical replicates). (g, h) Photography and DLS measurements of polymersomes with 10 mol % DMAEMA in different buffer environments: (i) pH 7.4 (HBSS buffer), (ii) pH 5.5 (acetate buffer), (iii) enzyme buffer with enzymes (50 nM thrombin) at pH 7.4, and (iv) AND gate sensing performed by first incubating in acetate buffer pH 5.5 for 5 min, then pH adjustment back to pH 7.4 by adding bicarbonate buffer pH 9.4 for 5 min, and finally adding 50 nM thrombin in enzyme buffer at pH 7.4 for a further 4-h incubation (mean value ± standard deviation, *n* = 3 technical replicates).

We first optimized the buffer for purification of polymersomes (5 mol % DMAEMA relative to HPMA), using 1× Dulbecco’s phosphate buffered saline (DPBS), 5× PBS, 250 mM phosphate buffer (PB) or 500 mM PB, all at pH 7.4. DLS was employed to measure the polymersome size and derived count rate (rough estimate of concentration at constant size) at pH 7.4 and 5.5 to establish stability at neutral pH and disassembly at acidic pH (Figure S8). 5X PBS was found to provide the best purification for long-term stability, while retaining the pH-responsive nature of the polymersomes. We also investigated 5, 10, and 15 mol % DMAEMA percentage (relative to HPMA) used during photo-PISA. Polymersomes with 10 mol % DMAEMA exhibited both a high stability at pH 7.4 and good pH responsiveness at pH 5.5 when analyzing storage over 4 days postsynthesis (Figure S9a–c). Importantly, TEM imaging confirmed that the polymersomes (containing 10 mol % DMAEMA) had sizes ranging from 300–500 nm (Figure 3b) and revealed the characteristic collapsed morphology expected for confirmation of polymersome structure.^50^ Polymersome formulations with different percentages of DMAEMA revealed neutral to slightly positive zeta potentials at a neutral pH (Figure 3c). Polymersome formulations with the optimized 10 mol % DMAEMA were cloudy at pH 7.4 and became transparent at pH 5.5 due to disassembly at low pH (Figures 3d and S9d), in agreement with our previous work.^36^ When incubated at 37 °C for up to 24 h, the polymersomes remained stable at neutral pH, while still responding to pH change by disassembly even after the incubation period (Figures 3e,f and S10).

To validate the pH-selective response of our platform, we subjected the pH-responsive polymersomes to different sensing conditions. Only low pH triggered disassembly, while thrombin alone showed no effect on the polymersome size (Figure 3g,h). The AND-gated platform involved incubation in acetate buffer at pH 5.5 for 5 min with pH adjusted back to 7.4 for 5 min using bicarbonate buffer (pH 9.4) and then adding thrombin (50 nM, in HBSS pH 7.4) for a 4-h incubation period at pH 7.4. This pH readjustment was necessary to restore optimal *in vitro* conditions for enzyme activity. The optimized polymersome formulation with 10 mol % DMAEMA was chosen for subsequent encapsulation of the nanosensor to form the final AND gate sensing platform.

### Formulation and Characterization of the AND Gate Sensing Platform

After optimizing and characterizing the nanosensor and the pH-responsive polymersomes, we loaded the THR and MMP-9 sensors, respectively, within the aqueous lumen of the pH-responsive polymersomes (called THR-Psome and MMP-Psome) by the simple addition of the nanosensors during polymersome synthesis. Centrifugation and resuspension were used for purification and after the second centrifugation step, no detectable TMB oxidation signal was observed in the supernatant, confirming effective removal of free nanosensors (Figure S11). DLS confirmed that the THR- and MMP-Psomes remained stable at neutral pH for 4 days (Figure S12), retained their pH responsiveness (Figure 4a,b) and exhibited neutral to slightly positive zeta potentials (Figure 4c). This also demonstrated that presence of AuNCs-NAv nanosensors did not inhibit the synthesis of polymersomes.

**Figure 4:**
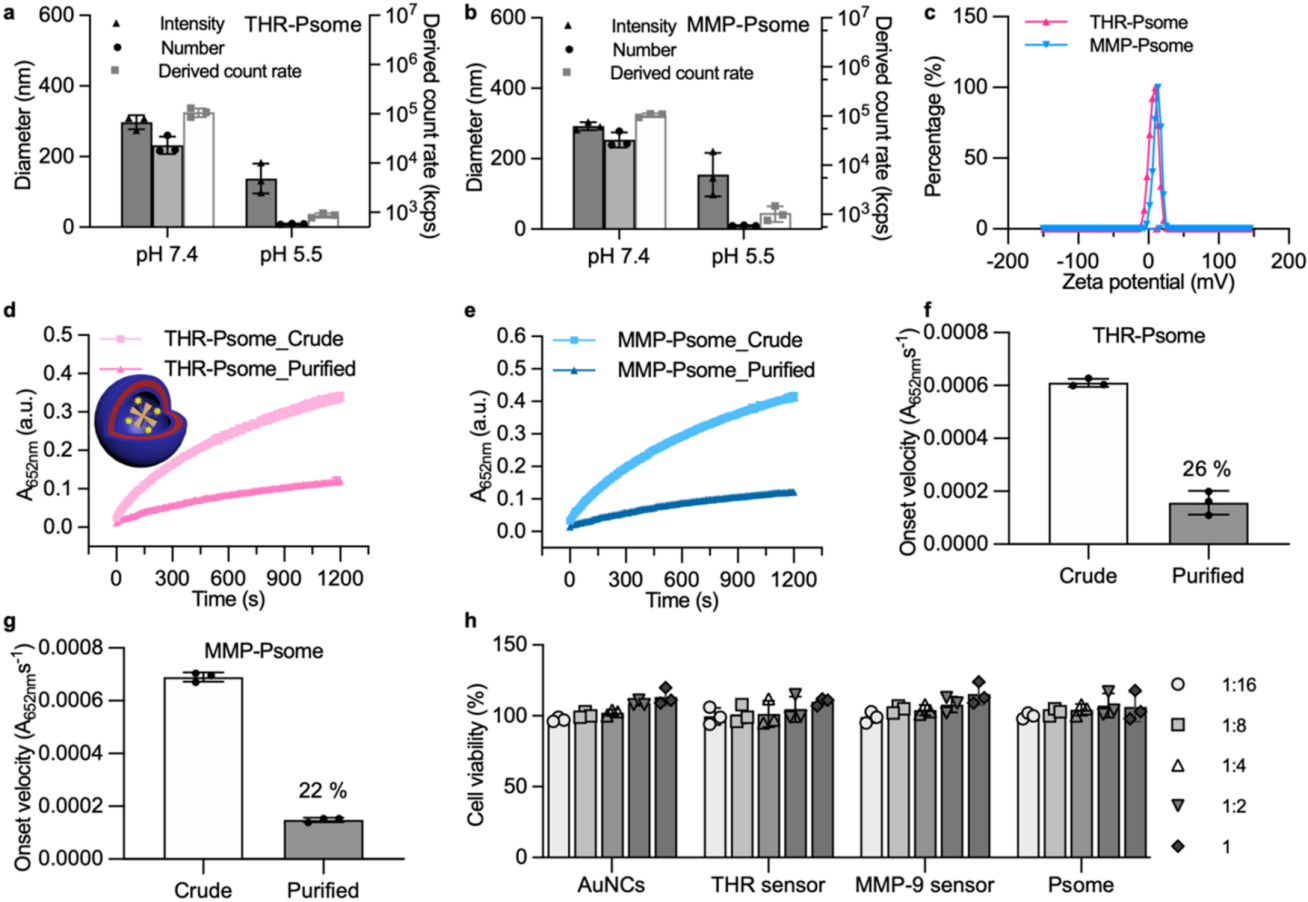
Characterization of THR sensor and MMP-9 sensor-loaded pH-responsive polymersomes (THR-Psome and MMP-Psome). (a, b) DLS measurements of (a) THR-Psome and (b) MMP-Psome in buffer pH 7.4 and 5.5 (mean value ± standard deviation, *n* = 3 technical replicates). (c) Zeta potential measurements of THR-Psome and MMP-Psome (mean of *n* = 3 technical replicates). (d, e) TMB kinetic absorbance measurements over time for (d) THR-Psome and (e) MMP-Psome. (f, g) TMB reaction onset velocity (from (d, e)) of (f) THR-Psomes and (g) MMP-Psome before and after purification. The encapsulation efficiency of the nanosensors was calculated by comparing onset velocity from purified and crude (unpurified) loaded PSomes (mean value ± standard deviation, *n* = 3 technical replicates). (h) *In vitro* cytocompatibility (MTS assay) with RAW 264.7 cells after incubation for 24 h with AuNC-GSH (1× = 20 μM), THR sensor (1× = 10 mg/mL, relative to NAv), MMP-9 sensor (1× = 10 mg/mL, relative to NAv) or empty polymersomes (1× = 10 mg/mL relative to PEG_113_-CDTPA) (mean value ± standard deviation, *N* = 3 biological replicates, *n* = 3 technical replicates).

Presence of the THR sensor and MMP-9 sensor in the pH-responsive polymersome was determined through TMB oxidation assays on both crude and purified AND-gated sensors (Figure 4d, e). Comparing the onset velocity of the catalytic reactions between crude and purified polymersomes yielded average encapsulation efficiencies of 26 and 22% for THR-Psome and MMP-Psome, respectively (Figure 4f, g). This agrees well with previously reported data for the *in situ* encapsulation of other proteins via photo-PISA,^36,51^ and it demonstrates that the photo-PISA reaction preserved the reactivity of the nanosensor even after purification.

To ensure future suitability of the system for *in vivo* sensing, we employed MTS assays using RAW 264.7 cells, which revealed a high biocompatibility of the system (Figure 4h). This agrees with previous literature, where AuNCs have previously shown good biocompatibility *in vitro* and *in vivo*,^9,10,52^ NAv is biocompatible,^53,54^ while PEGylated polymersomes of this composition are also known for their biocompatibility.^36,55^ In conclusion, we demonstrated the synthesis of a biocompatible platform that consists of enzyme-responsive nanosensor loaded in pH-responsive polymersomes.

### Sensing Performance of AND-Gated Diagnostic Platform

Finally, we investigated whether the sensing platform could respond to both low pH conditions and then a specific enzyme to generate an AND logic gate operation. To validate this capability, four experimental conditions were designed: (i) pH 7.4 (HBSS buffer), (ii) pH 5.5 (acetate buffer), (iii) enzyme buffer with enzymes (either 50 nM thrombin or MMP-9) at pH 7.4, and (iv) AND gate sensing performed by first incubating in acetate buffer pH 5.5 for 5 min, then pH adjustment back to pH 7.4 by adding bicarbonate buffer pH 9.4 for 5 min, and finally adding 50 nM enzyme in enzyme buffer at pH 7.4 for a further 4 h incubation (Figure 5a). The AuNCs should be cleaved from the nanosensor only upon exposure of the sensing platform to an acidic environment (triggering polymersome disassembly), followed by enzyme treatment (iv). The liberated AuNCs are then separated using centrifugal filtration, followed by a TMB oxidation assay, which should result in a visible blue color change.

**Figure 5:**
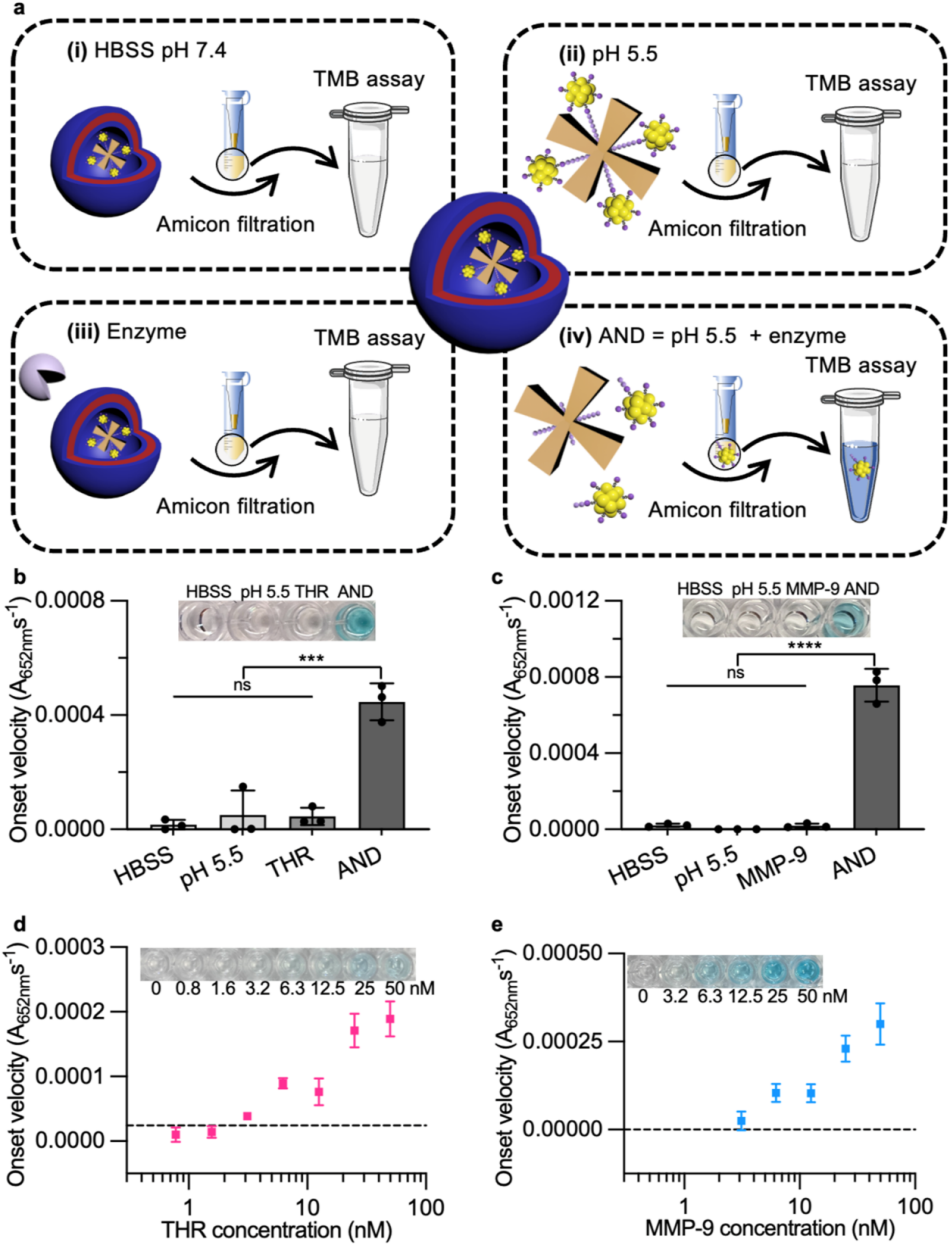
AND gate responsive sensing system for detection of low pH and a specific enzyme. (a) Schematic of AND gate sensor in four different conditions: (i) pH 7.4 (HBSS buffer), (ii) pH 5.5 (acetate buffer), (iii) enzyme buffer with enzymes (either 50 nM thrombin or MMP-9) at pH 7.4, and (iv) incubating in acetate buffer pH 5.5 for 5 min, then pH adjustment back to pH 7.4 by adding bicarbonate buffer pH 9.4 for 5 min, and finally adding 50 nM enzyme in enzyme buffer at pH 7.4 for a further 4 h incubation. (b, c) TMB assay on filtrates after incubation of (b) THR-Psome and (c) MMP-Psome in four different conditions (mean value ± standard deviation, *N* = 3 biological replicates, *n* = 3 technical replicates, one-way ANOVA with Tukey’s multiple comparisons test, *** *p* < 0.001 for panel (b), **** *p* < 0.0001 for panel (c) and ns: not statistically significant *p* > 0.05). (d, e) *In vitro* cleavage performance of (d) THR-Psome and (e) MMP-Psome in the AND gate sensing condition shown in panel (a, (iv)) (mean value ± standard deviation, *N* = 3 biological replicates, *n* = 3 technical replicates). The dashed line indicates the LoD, derived from the mean background signal plus 3 standard deviations. Figure 5b–e display onset velocity as the primary readout. Equivalent data using end point absorbance (*A*_652 nm_ at 10 min) are provided in Figure S14, showing similar consistent results.

The four experimental conditions were applied to two types of AND-gated sensors: THR-Psome (responsive to both pH 5.5 and thrombin) and MMP-Psome (responsive to both pH 5.5 and MMP-9), using thrombin and MMP-9 as the respective enzymes. As hypothesized, a blue color change was only observed in samples subjected to both low pH < 6.5 and specific enzyme treatment, confirming the AND gate sensing capability. No color change was detected in any of the other three conditions (Figure 5b,c). It demonstrated that enzymes cannot access the polymersome at neutral pH, and acidic conditions alone are insufficient to cleave the NAv complex into fragments below the molecular weight cutoff of centrifugal filters. The absorbance at 652 nm over time during TMB oxidation exhibited a similar absorbance increase for both THR-Psome and MMP-Psome sensors when treated with pH 5.5 and thrombin/MMP-9, while maintaining a low background under other conditions (Figure S13). This demonstrated that the final sensor platform effectively acts as an AND gate for a low pH environment and a specific enzyme.

The LoD values for both THR-Psome and MMP-Psome sensors were then determined. The LoDs of the THR-Psome and MMP-Psome sensors were approximately 2.5 and 2 nM respectively, derived from the mean background signal plus 3 standard deviations (Figure 5d,e), demonstrating that both THR-Psome and MMP-Psome reached the same LoDs of the nanosensor alone (Figure 2c,d) but now with the additional pH trigger that is needed for the initial liberation of the nanosensor from the polymersomes. These results confirm the AND-gate behavior of the sensing platform in the presence of both pH 5.5 and enzyme, leading to significant signal generation (Figure 5). To address clinical translation, we also analyzed end point absorbance (*A*_652 nm_ at 10 min). The results closely mirror the onset velocity data, demonstrating that a simple single-time point absorbance readout could also be used, which would be particularly suitable for resource-limited settings or naked-eye detection (Figure S14). This *in vitro* AND gate sensing platform demonstrates a robust sensing platform capable of detecting two biochemical stimuli, thereby enhancing the specificity of the sensing performance. While dual-responsive and logic-gated biosensors can enhance specificity for diagnostics and therapy, many reported systems require complex instrumentation or advanced imaging devices for detection, underscoring the need for modular, clinically practical platforms with simple, naked-eye readability, especially for resource-limited settings.^6,7,31,41–46^ To this end, our platform integrates catalytically active AuNCs, which are ultrasmall, biocompatible, and renally clearable, with orthogonal and modular PEGylated stimuli-responsive polymersomes synthesized by photo-PISA. This combination yields a modular, stable, and highly specific sensing system capable of a simple colorimetric readout, thereby enhancing translational potential compared with existing designs. Due to the precise size changes from ∼ 300–500 nm, to ∼10 nm, to ∼2 nm upon exposure to the two stimuli, we envision potential future *in vivo* usability of such a system. After the last step (enzymatic cleavage), the ultrasmall detection units (AuNCs) could be excreted via the kidneys to be measured with a simple colorimetric readout in the urine, as we have previously demonstrated with other sensors.^9,10^

Although the present study employed physiological buffer conditions to characterize the AND-gated platform, both individual core components, the peptide-templated AuNCs and PEGylated pH-responsive PISA-derived polymersomes, have demonstrated high colloidal and catalytic stability in previous *in vitro* and *in vivo* studies upon exposure to physiological environments, including different pH conditions mimicking tumor or inflammatory microenvironment.^9,10,36^ In particular, the same AuNCs have been shown to retain colloidal stability and catalytic activity in serum and urine, which enabled colorimetric detection in urine after renal clearance.^9,10^ PISA-derived PEGylated polymersomes are well-known for their resistance to nonspecific protein adsorption and prolonged stability in blood,^55,56^ while the formulation we have used herein has previously shown high stability in neutral PBS (pH 7.4) and strong capability for the controlled release and delivery of loaded cargos.^36^ Together, these features support the anticipated robustness of our dual-responsive system in complex biological fluids, with detailed evaluation of the coassembled system planned for future work. We envision that the presented AND gate sensor could be administered *in vivo* and report on the condition of acidic pH and enzymatic activity through a simple colorimetric urinary readout, making it highly suited for translational applications such as noninvasive disease monitoring. Overall, these results highlight the potential of this AND logic gate sensing platform for precise and selective detection in biomedical applications.

## Conclusions

This AND logic gate sensing platform represents an advancement in diagnostic sensors by applying a versatile hierarchical approach with the potential to improve the precision and specificity of disease diagnostics by suppressing background signals and reducing false positives from single-stimulus activation. By integration of a pH-responsive polymersome with an enzyme-activated nanosensor, the system triggers a detectable signal only when both stimuli are present, making it particularly useful for distinguishing complex disease states. Potential applications of this system are in cancer diagnostics and inflammatory diseases such as rheumatoid arthritis, where acidic microenvironments and elevated enzyme levels, such as MMPs, are common.^57–59^ Although this proof-of-concept was conducted under buffer conditions, both sensing components, the polymersome formulation and the AuNC complex, have previously shown high stability *invivo*,^9,10,36^ supporting the potential for future translation of this dual-responsive sensor into physiological environments. A key advantage of this sensor design over other logic-gated biosensors is its modularity: the AuNC can be functionalized with diverse peptide templates to target a range of protease or enzyme substrates, enabling versatile adaptation to specific disease contexts. We envision this sensor could be administered *in vivo*, where pH-responsive disassembly and enzyme-mediated cleavage would occur locally within acidic and proteolytic tumor or inflammatory environments. The liberated ultrasmall AuNCs could then be cleared renally and detected noninvasively in urine, making the system particularly suited for resource-limited settings due to the simple colorimetric readout by the naked eye. This innovative system underscores the potential of materials science in developing accessible, precise, and adaptable diagnostic tools that can sense and respond to multiple environmental signals for a wide range of diagnostic applications.

## Experimental Section

### Materials

Gold chloride trihydrate (HAuCl_4_), l-glutathione reduced (GSH), biotin, NeutrAvidin Protein (NAv), 3,3′,5,5′-Tetramethylbenzidine (1-Step Ultra TMB-ELISA Substrate Solution), 30% (w:w) hydrogen peroxide solution (H_2_O_2_), thrombin from human plasma, recombinant human MMP-9, poly­(ethylene glycol) monomethyl ether (mPEG_113_, Mn = 5000 g/mol), *N*,*N*′-dicyclohexylcarbodiimide (DCC), 4-(dimethylamino)­pyridine (DMAP), 2-hydroxypropyl methacrylate (HPMA), 2-(dimethylamino)­ethyl methacrylate (DMAEMA), mineral oil (BioReagent), sodium phosphate dibasic heptahydrate, sodium phosphate monobasic monohydrate, Hanks′ balanced salt solution (HBSS), and Amicon ultra centrifugal filter (10 and 30 kDa MWCO, 15 and 0.5 mL) were purchased from Sigma-Aldrich. 4-Cyano-4-[(dodecylsulfanylthiocarbonyl)­sulfanyl] pentanoic acid (CDTPA) was purchased from Boron Molecular. 2-Chlorotrityl chloride resin was purchased from Iris Biotech. The synthesis of PEG_113_-CDTPA was performed following a published literature.^47^ HPMA was purified using silica column chromatography with ethyl acetate/hexane (1:9) as eluent to remove the impurities, while DMAEMA was purified to remove the inhibitor using a column containing basic aluminum oxide. Dulbecco’s phosphate buffered saline (DPBS), Pierce biotin quantitation kit, acetate buffer (1 M, pH 5.5), HEPES buffer (pH 7.4), DMEM without phenol red (1×, high glucose, GlutaMAX, Gibco, 31966-021), fetal bovine serum (FBS), and penicillin-streptomycin (1000 U mL^–1^) were purchased from Thermo Fisher Scientific. RAW 264.7 cells were obtained from ATCC. MTS (3-(4,5-dimethylthiazol-2-yl)-5-(3-carboxymethoxyphenyl)-2-(4-sulfophenyl)-2H-tetrazolium) assay (Promega) was purchased from Abcam. Amino acid, *N*,*N*-diisopropylethylamine (DIPEA), piperidine, dichloromethane (DCM), dimethylformamide (DMF), hexafluorophosphate azabenzotriazole tetramethyl uronium (HATU), trifluoroacetic acid (TFA), triisopropylsilane (TIS), dithiothreitol (DTT), and acetonitrile were used as purchased. Milli-Q water (18.2 MΩ cm) was used in all experiments.

### Synthesis of Peptide and Characterization

The solid-phase peptide synthesis method was used for our targeted peptide synthesis (thrombin-cleavable peptide: biotin-SGGfPRSGGSGGC and MMP-9-cleavable peptide: biotin-GGGPLGVRGKGGC from a previous study^10^), utilizing standard fluorenyl methoxycarbonyl (Fmoc) chemistry for the peptide synthesis on 2-chlorotrityl chloride resin (Table S1 and Figure S1). The targeted peptides contain a C-terminal carboxylic acid protected with Fmoc, which undergoes esterification with the 2-chlorotrityl chloride functional group. The first C-terminal Fmoc-protected amino acid (3 equiv) was added to the resin along with DIPEA (6 equiv) in a DMF/DCM (50:50 v/v) mixture (3 mL for 0.5 g of resin) and shaken overnight. The solution was then expelled, and the resin was washed with DCM (5 × 5 mL), followed by the addition of a capping solution containing methanol (1 mL), DIPEA (0.5 mL), and DCM (9 mL), with shaking for 20 min to cap unreacted sites. The washing was subsequently performed with DCM and DMF (each 5 × 5 mL for 0.5 g of resin). Fmoc deprotection was carried out to remove the Fmoc group from the amino acid before the next amino acid coupling. This was achieved by incubating the resin with a piperidine/DMF solution (20:80 v/v, 5 mL for 0.5 g of resin) twice for 10 min each. The resin was then washed with DMF (5 × 5 mL) before coupling to the next amino acid. For coupling, the next Fmoc-protected amino acid (3 equiv) was dissolved in HATU solution (0.5 M, 2.95 equiv in DMF), with the addition of DIPEA (6 equiv), and the mixture was shaken for 15 min for activation before coupling. The activated solution was then added to the resin and shaken for 1–3 h. The resin was washed with DMF (5 × 5 mL) before the next deprotection step. The required amino acids and final biotin were prepared and coupled by repeating the deprotection and coupling steps, as mentioned above. After completing the full sequence, the resin was washed with DCM (5 × 5 mL) and dried under a vacuum. Cleavage was performed using a solution of TFA (87.5% v/v), TIS (2.5% v/v), DTT (7.5% w/v), and water (2.5% v/v), typically 7 mL for 0.5 g of resin, and shaking for 3 h. The solution was then expelled into a new round-bottom flask, and solvents were removed by using a rotary evaporator to obtain the peptide. The crude peptide was dissolved in water containing 0.1% v/v TFA and 0.1% v/v acetonitrile, filtered by using a PTFE frit, and purified by HPLC. LC-MS was used to verify the mass and purity of the peptide, and the correct peptide fractions were collected for freeze-drying, making them ready for use.

### Peptide-Templated AuNC-GSH Synthesis

Peptide-templated AuNCs were synthesized and purified following published procedures, with the modifications outlined below.^10^ The percentage of protease-cleavable peptide was varied in the ligand composition for reducing and capping on the AuNC surface (20, 5, 2, and 1 mol %). Briefly, an aqueous solution of HAuCl_4_ (20 mM, 100 μL) was added to water (750 μL) in an Eppendorf tube, followed by the rapid addition of GSH (20 mM) and peptide (20 mM), both at a fixed volume of 150 μL but with various peptide mole percentages in ligand composition. The reaction mixture was heated to 70 °C with shaking at 500 r.p.m. for 24 h. After the 24-h incubation, peptide-templated AuNCs were purified and buffer-exchanged with DPBS using Amicon Ultra-15 Centrifugal Filter Units (MWCO 10 kDa) at 5000*g* for 15 min, repeated three times. Finally, the AuNCs were suspended in DPBS to yield a final concentration of 20 μM and subsequently filtered through a sterile syringe filter (0.22 μm Millex-GV Filter, Millipore) into a new Eppendorf tube. The purified AuNCs were stored at 4 °C for over six months for further use.

### Assembly of THR and MMP-9 Responsive Nanosensor and the Cleavage Assay

Typically, NAv (8 mg/mL in DPBS, 125 μL) was added to peptide-templated (thrombin-cleavable peptide or MMP-9-cleavable peptide) AuNCs (20 μM, 1 mL in DPBS) and incubated at 37 °C with gentle shaking at 500 rpm for 3 h. Centrifugal ultrafiltration was then performed to remove unbound AuNCs from the complex nanosensors (THR sensor or MMP-9 sensor) using Amicon Ultra-15 centrifugal filter units (MWCO 30 kDa, Sigma). Finally, the nanosensors were resuspended in DPBS and sterile-filtered through a Millex-GV filter (0.22 μm, Millipore).

For the cleavage assay, the purified primary sensors (80 μL, 1 mg/mL for NAv in DPBS) were incubated separately with thrombin or MMP-9 (80 μL, 50 nM in DPBS) at 37 °C for 3 h under gentle shaking at 300 r.p.m. After incubation, the solutions were centrifuged using Amicon Ultra-0.5 Centrifugal Filter Units (MWCO 30 kDa) at 14 000*g* for 15 min. The filtrates, containing liberated AuNCs, were collected for the TMB assay. The TMB oxidation assay was performed by mixing 10 μL of filtrate with a mixture of a 1-Step Ultra TMB-ELISA Substrate Solution (45 μL) and H_2_O_2_ (45 μL, 10 M). Absorbance was measured at 652 nm using a SpectraMax M5 multimodal microplate reader. The LoD for thrombin and MMP-9 cleavage was determined by incubating the nanosensors with a serial dilution of the enzyme (ranging from 0 to 50 nM) for 3 h, followed by TMB assay in the filtrate after centrifugation, which was determined as 10% of the maximum absorbance signal from fits using a four-parameter logistic equation.

### Photo-PISA Synthesis of Polymersomes and *In Situ* Sensor Encapsulation

Polymersome synthesis followed a recently published protocol.^36^ Briefly, PEG_113_-CDTPA was dissolved in methanol at a concentration of 10 mg/mL as a stock solution. The stock solution (113.8 μL, 0.21 μmol) was added to a low-protein-binding Eppendorf tube, and the solvent was dried overnight at room temperature. HPMA (80.1, 75.9, or 71.7 μmol) and DMAEMA (4.2 μmol for 5 mol % monomer content, 8.43 μmol for 10 mol % monomer content, 12.6 μmol for 15 mol % monomer content; 93.3 mg/mL in 100 mM HEPES buffer) were added to the dried PEG_113_-CDTPA pellet, and the volume was adjusted to 100 μL with 100 mM HEPES buffer (pH 7.4). The prepared solutions (100 μL) were transferred to a 384-well plate and covered with 5 μL of mineral oil to prevent evaporation during polymerization. The plate was irradiated with a 405 nm LED array for 3 h to initiate the photo-PISA reaction. The resulting crude polymersomes were collected and diluted with 5X PBS to a total volume of 1 mL, followed by centrifugation at 14 000*g* for 10 min at room temperature. The supernatant was gently discarded, and the pellet was resuspended in 1 mL of 5× PBS. This washing step was further repeated twice. The final pellet was collected and stored for further analysis.

The thrombin-responsive and MMP-9-responsive AuNC-NAv sensors were assembled as described in the above section. Finally, they were buffer exchanged into 100 mM HEPES buffer with a final NAv concentration of 10 mg/mL for reference. The loading of nanosensors into polymersomes followed the standard polymersome synthesis procedure described above. Briefly, HPMA and DMAEMA were added to PEG_113_-CDTPA, followed by topping up to 100 μL with the nanosensors prepared above. THR sensor-loaded pH-responsive polymersomes (THR-Psomes) and MMP-9 sensor-loaded pH-responsive polymersomes (MMP-Psomes) were prepared by following the sensor-loaded polymersome procedure.

### Characterization of Single-Nanosensor and AND-Gated Sensing Platform

Peptide density (peptides per AuNC) was quantified by using Pierce biotin quantitation kit measuring the number of biotinylated ligands (i.e., peptide) in the filtrate from AuNC purification. The biotin or peptide concentration was quantified following the instruction from Thermo Fisher by subtracting the amount in the filtrate from the starting concentration. The hydrodynamic diameter (dynamic light scattering, DLS) and zeta potential of AuNCs, the nanosensors, and dual-responsive polymersome sensors were measured using a Zeta Sizer Nanoseries (Malvern Instruments, Ltd.). The kinetic reaction of TMB oxidation by AuNC catalysts, the nanosensor, and AND gate responsive sensor was monitored with a SpectraMax M5 multimodal microplate reader. The TMB oxidation assay was utilized for the comparison of the catalytic activity, using a 1:1 v/v mixture of a 1-Step Ultra TMB-ELISA substrate solution and 10 M H_2_O_2_ (pH = 3), unless specified otherwise. The catalytic activity was measured by calculating the onset velocity, through the linear curve at the onset of the kinetic reaction of the absorbance over time (*A*_652 nm_ s^–1^). UV–vis was used for measuring the contents of AuNCs for the verification of AuNC formation and related nanosensors. Transmission electron microscopy (TEM) images of AuNC-NAv and polymersomes were obtained on a JEOL 2100F. To prepare samples for TEM, an AuNC-NAv solution was desalted first using Zeba Spin Desalting Columns (7 K MWCO). Five μL of desalted solution or polymersome was dropped on an individual carbon-coated copper grid drying overnight for imaging.

### Cytotoxicity Test

RAW 264.7 cells were cultured using culture media consisting of DMEM 1× medium with 10% (v/v) FBS and 0.5% v/v penicillin-streptomycin. For seeding cells, the desired concentration of RAW 264.7 cells in culture media were prepared and transferred into sterile 96-well plates (20 000 cells/well), followed by incubation for 24 h for adhesion. The culture media from the wells with the nonadherent RAW 264.7 cells was removed. Twenty μL of AuNC-GSH, THR sensor, MMP-9 sensor, and polymersome synthesized with 10 mol % DMAEMA and all relative dilution in HEPES (pH 7.4) were added in the wells with an additional 180 μL of medium. The 96-well plates were incubated at 37 °C with 5% CO_2_ for 24 h. The cell viability assay was conducted using MTS assay (MTS) by discarding the cell supernatants (200 μL) and replacing with 120 μL of MTS stock solution containing DMEM medium without phenol red (31.5 mL), MTS (2 mg/mL, 6 mL), PMS (0.92 mg/mL, 0.3 mL), incubation for 1 h, and measurement of absorbance at 490 nm.

### AND Gate Sensing Assay of THR-Psome and MMP-Psome

A total of 100 μL of THR-Psome or MMP-Psome was divided into four 25 μL aliquots, which were then centrifuged at 14 000*g* for 15 min to obtain pellets. Each pellet was resuspended in one of the following conditions: (i) pH 7.4 (1× HBSS buffer, 100 μL), (ii) pH 5.5 (100 mM acetate buffer, 100 μL), (iii) enzyme buffer with enzymes (either 50 nM thrombin or MMP-9 in HBSS with 10 μM Zn^2+^, 100 μL) at pH 7.4, and (iv) AND gate sensing performed by first incubating in acetate buffer pH 5.5 (100 mM, 75 μL) for 5 min, then pH adjustment back to pH 7.4 by adding bicarbonate buffer pH 9.4 (100 mM, 10 μL) for 5 min, and finally adding enzyme in enzyme buffer (either 50 nM thrombin or MMP-9 in HBSS with 10 μM Zn^2+^, 15 μL) at pH 7.4 for a further 4-h incubation. After incubation, Amicon Ultra-0.5 centrifugal filter units (MWCO 30 kDa, Sigma) were used to separate the released AuNCs for TMB oxidation assays from the filtrates. The assays were performed by mixing 25 μL of filtrate with a mixture of 1-Step Ultra TMB-ELISA substrate solution (100 μL) and H_2_O_2_ (100 μL, 10 M) and absorbance was measured at 652 nm over time using a SpectraMax plate reader. The LoD was determined by incubating the sensors with a serial dilution of the enzyme (ranging from 0 to 50 nM) for 4 h, followed by TMB assay in the filtrate after centrifugation, which was estimated through the baseline that could be measured and derived from the mean background signal plus 3 standard deviations.

## Supplementary Material



## Notes

Detailed description of protease-cleavable peptide sequences, conjugation efficiency of nanosensors, and characterization of AuNC-based enzyme-responsive sensors (UV–vis, HABA assay, DLS, zeta potential, TEM, and TMB oxidation assays); preparation and characterization of pH-responsive polymersomes under various buffer and storage conditions; encapsulation and purification of thrombin- and MMP-9-responsive sensors within polymersomes; and performance evaluation of the resulting stimuli-responsive polymersomes for AND logic gate sensing under different environmental conditions (PDF)

## §

J.Y.: Graduate School of Biomedical Engineering, University of New South Wales, Sydney 2052, New South Wales, Australia
