# Supplementary material for: Photo-PISA Driven In Situ Encapsulation of Nanocluster-Based Sensors within Stimuli-Responsive Polymersomes for AND Logic Gate Sensing
Source: ACS Sens. 2025 Nov 2;10(11):8778–89. doi: 10.1021/acssensors.5c02685 (PMC12670991; doi:10.1021/acssensors.5c02685)
Supplement: Supplementary file 1 [file se5c02685_si_001.pdf]

Supporting information

Photo-PISA Driven In Situ Encapsulation of Nanocluster-Based Sensors within Stimuli-Responsive Polymersomes for AND Logic Gate Sensing

Kaili Chen[1], Colleen N. Loynachan[1], Chalaisorn Thanapongpibul[1], Junni Zhang[1,2], Liyun Ma[1,2], Jonathan Yeow[1*], Adrian Najer[1*], Molly M. Stevens[1,2*]

[1]Department of Materials, Department of Bioengineering, and Institute of Biomedical Engineering, Imperial College London, SW7 2AZ, London, United Kingdom

[2]Department of Physiology, Anatomy and Genetics, Department of Engineering Science, Kavli Institute for Nanoscience Discovery, University of Oxford, OX1 3QU, Oxford, United Kingdom

Corresponding author emails: j.yeow@unsw.edu.au, a.najer@imperial.ac.uk, molly.stevens@dpag.ox.ac.uk

Table of Contents

Table S1	S3
Table S2	S3
Figure S1	S4
Figure S2	S5
Figure S3	S6
Figure S4	S7
Figure S5	S8
Figure S6	S9
Figure S7	S9
Figure S8	S10
Figure S9	S11
Figure S10	S12
Figure S11	S13
Figure S12	S14
Figure S13	S14
Figure S14	S15
References	S16

Table S1. Protease-cleavable thiol- and biotin-terminated peptide sequences for the assembly of enzyme-responsive nanosensors.[1]

Substrate	Protease specificity	Sequence	MW (g/mol)
P1	Thrombin	Biotin-SGG**fPR**SGGSGGC	1350
P2	MMP-9	Biotin-GGG**PLG**VRGKGGC	1339

Table S2. The conjugation efficiency for THR and MMP-9 nanosensors with different percentage of peptide used during AuNC synthesis.

	Peptide concentration in ligand contents (%)			
% Conjugation efficiency	20	5	2	1
THR nanosensor	0.5%	18.7%	9.2%	4.0%
MMP nanosensor	2.6%	16.2%	8.1%	3.0%

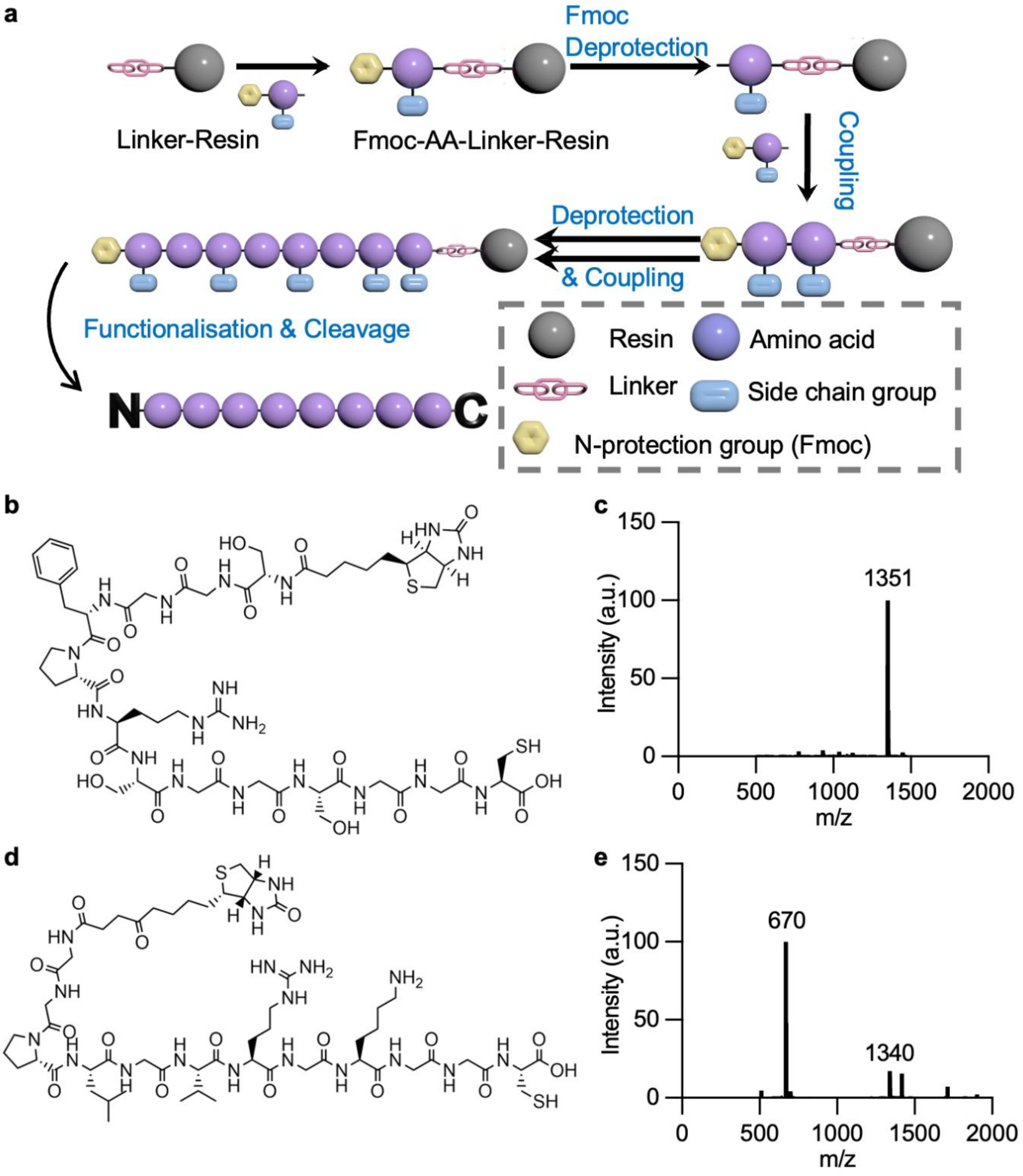

Figure S1. Solid phase peptide synthesis schematic and characterization of peptides. a. Solid phase peptide synthesis (SPPS) mechanism. **b, c.** Chemical structure and liquid chromatography-mass spectrometry analysis of thrombin responsive peptide (P1). **d, e.** Chemical structure and liquid chromatography-mass spectrometry analysis of MMP-9 responsive peptide (P2).

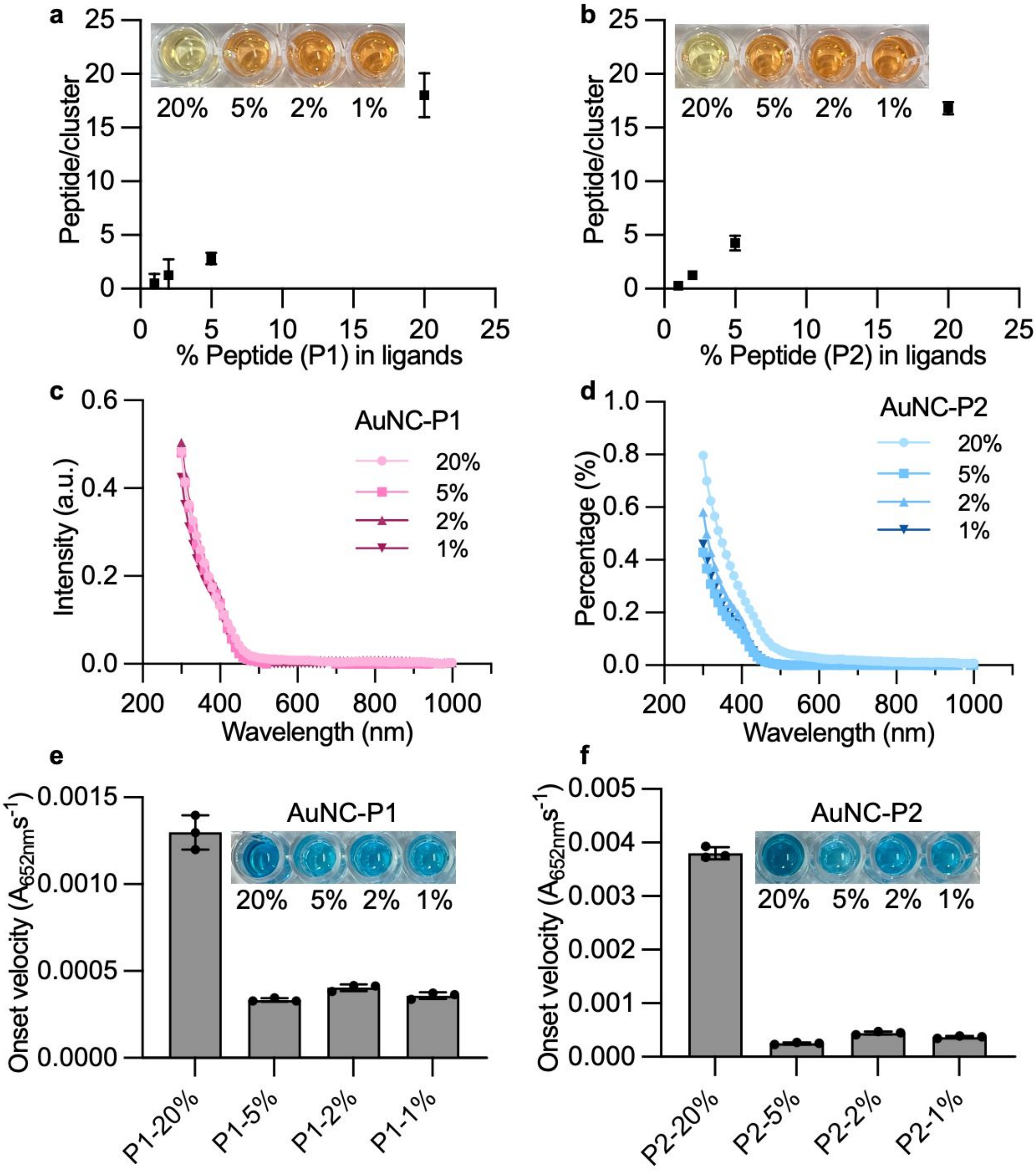

Figure S2. HABA assay and characterization of AuNCs templated with different peptide percentage. a, b. HABA assay quantifying the amount of biotin per AuNC for (**a**) thrombin-cleavable peptide (P1) and (**b**) MMP-9-cleavable peptide (P2) (mean values ± standard deviation, n = 3 technical replicates). **c, d.** UV-vis measurement of (**c**) AuNC-P1 and (**d**) AuNC-P2 templated with different peptide percentage (n = 1 technical measurement). **e, f.** Catalytic activity of (**e**) AuNC-P1 and (**f**) AuNC-P2 templated with different peptide percentage (mean values ± standard deviation, n = 3 technical replicates).

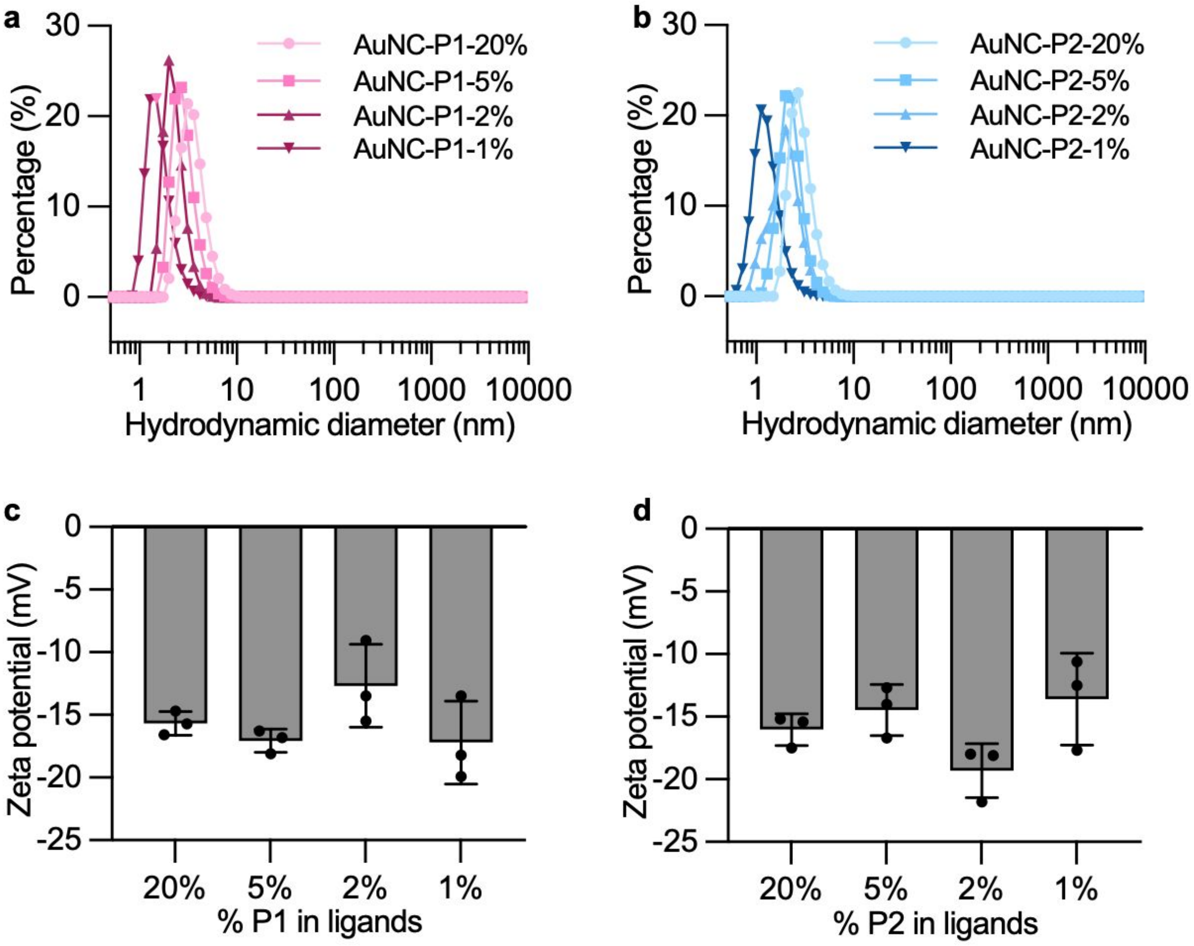

Figure S3. DLS and zeta potential characterization of AuNC-P1 and AuNC-P2. **a, b.** DLS number-derived size distribution of (**a**) AuNC-P1 and (**b**) AuNC-P2 synthesized with different peptide percentage (curves are mean of n = 3 technical measurements). **c, d.** Zeta potential measurements of AuNC-P1 (**c**) and AuNC-P2 (**d**) (mean values ± standard deviation, N = 3 Individual replicates, n = 1 technical replicate).

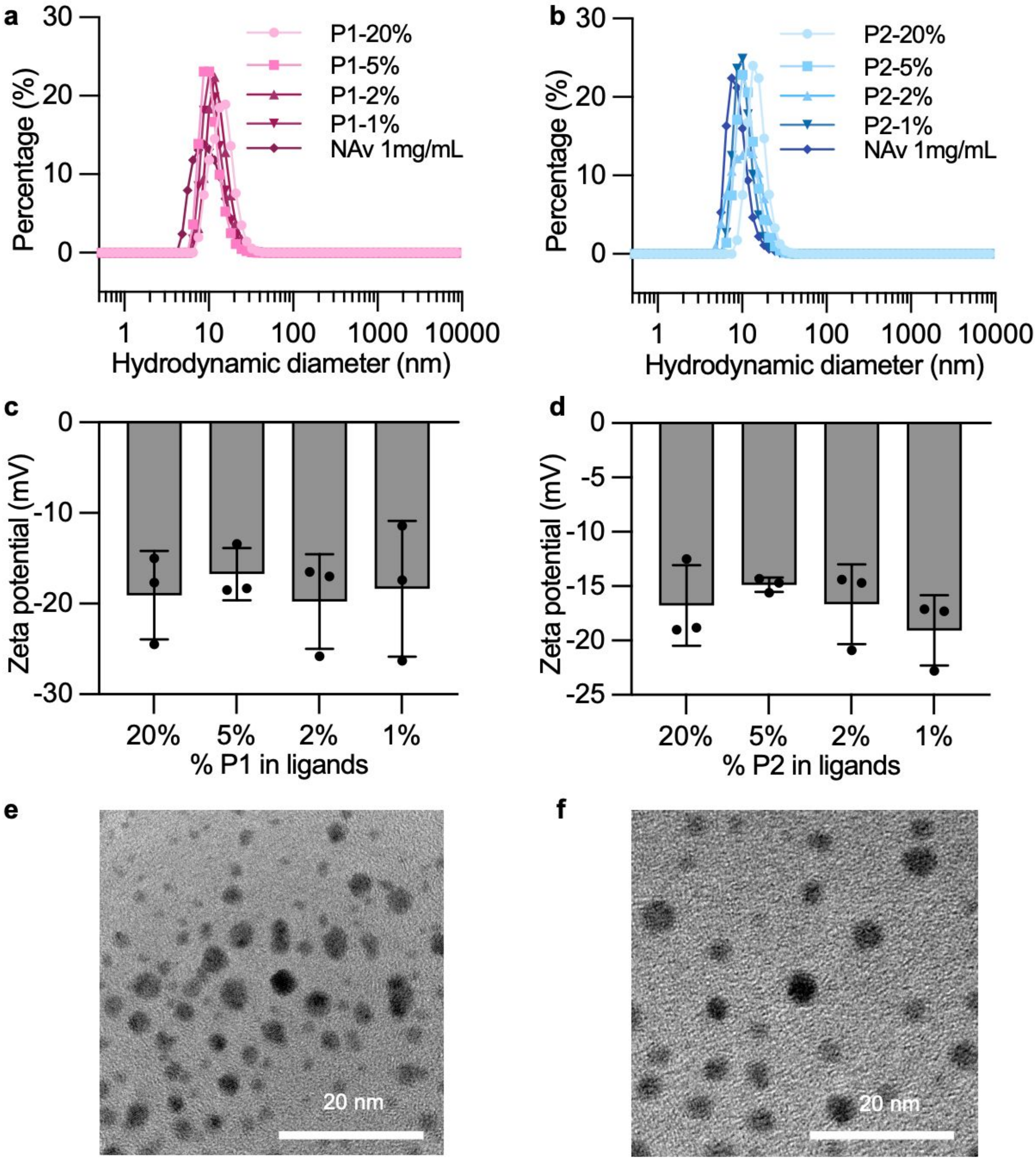

Figure S4. Characterization of THR sensor and MMP-9 sensor. a, b. DLS number-derived size distribution of (**a**) THR sensor and NAv and (**b**) MMP-9 sensor and NAv with different percentage of peptides (curves are mean of n = 3 technical measurements) **c, d.** Zeta potential measurement of (**c**) THR sensor and (**d**) MMP-9 sensor (mean values ± standard deviation, N = 3 Individual replicates, n = 3 technical replicates). **e, f.** TEM images of THR sensors (a total of 18 images were taken with similar results) and MMP-9 sensors (a total of 33 images were taken with similar results). Scale bar is 20 nm.

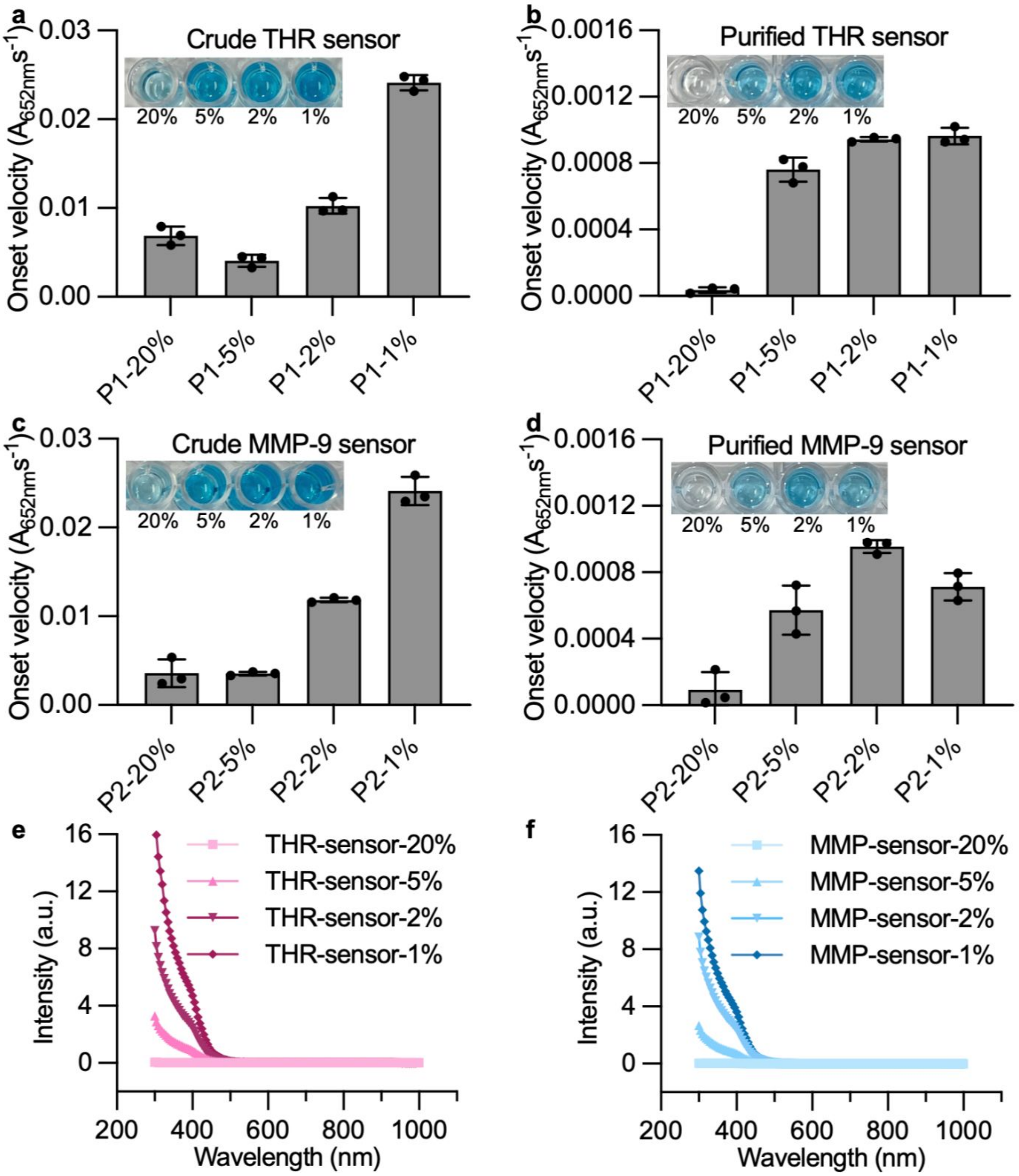

Figure S5. TMB oxidation assay of THR sensor and MMP-9 sensor. a-d. Onset velocity from TMB oxidation assays for (**a**) crude and (**b**) purified THR sensor, and (**c**) crude and (**d**) purified MMP-9 sensor (mean values ± standard deviation, N = 3 Individual replicates, n = 1 technical replicate). **e, f.** UV-vis measurement of (**e**) THR sensor and (**f**) MMP-9 sensor with different peptide percentage during AuNC synthesis (n = 1 technical measurement).

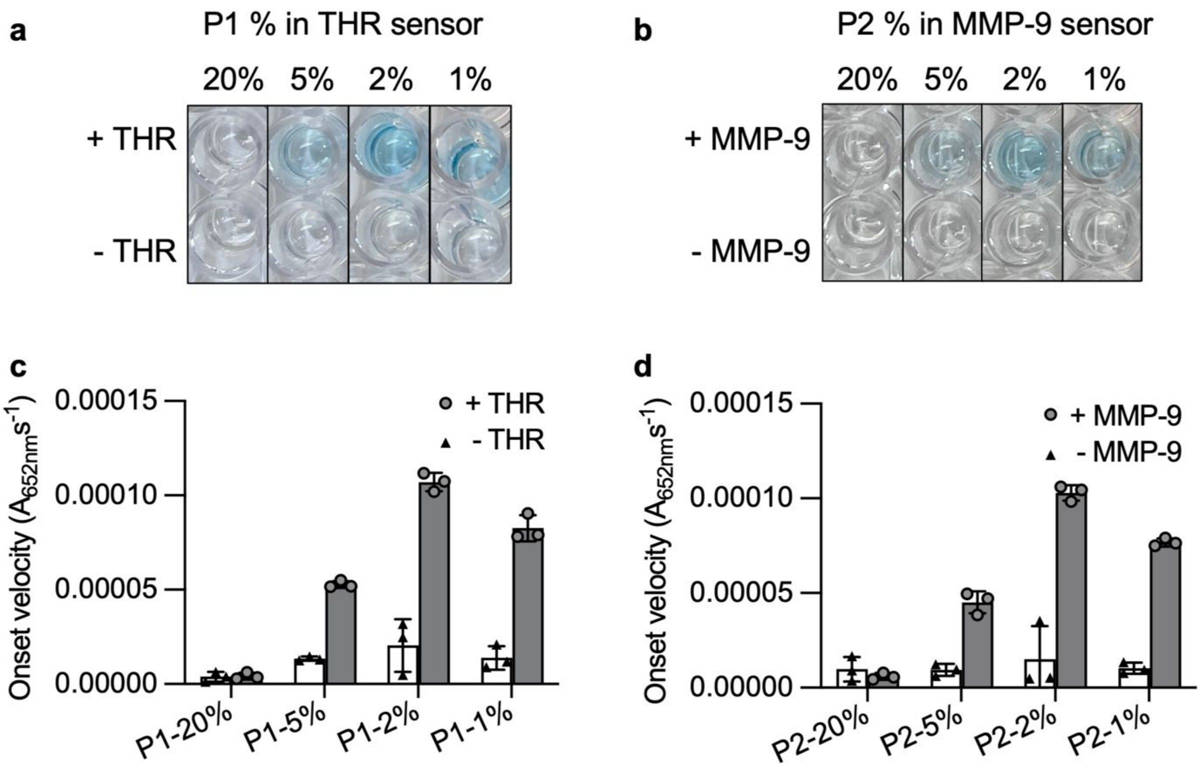

Figure S6. Enzymatic cleavage performance of THR sensor and MMP-9 sensor. **a, b.** Photographs of sensing performance of (**a**) THR sensor and (**b**) MMP-9 sensor with and without addition of the specific enzyme, measuring the filtrate after centrifugal filtration. **c, d.** Corresponding onset velocity from TMB oxidation assays on filtrates, same as **a, b** (mean values ± standard deviation, n = 3 technical replicates).

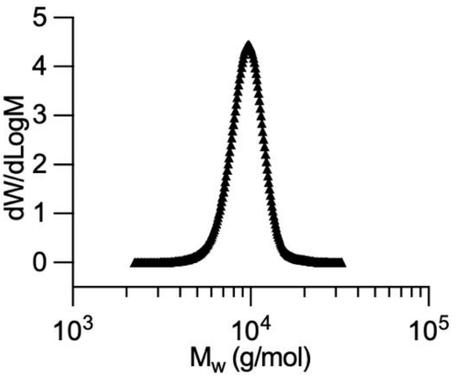

Figure S7. GPC trace of polymer initiator. Molecular weight distribution of purified PEG_{113}-CDTPA.

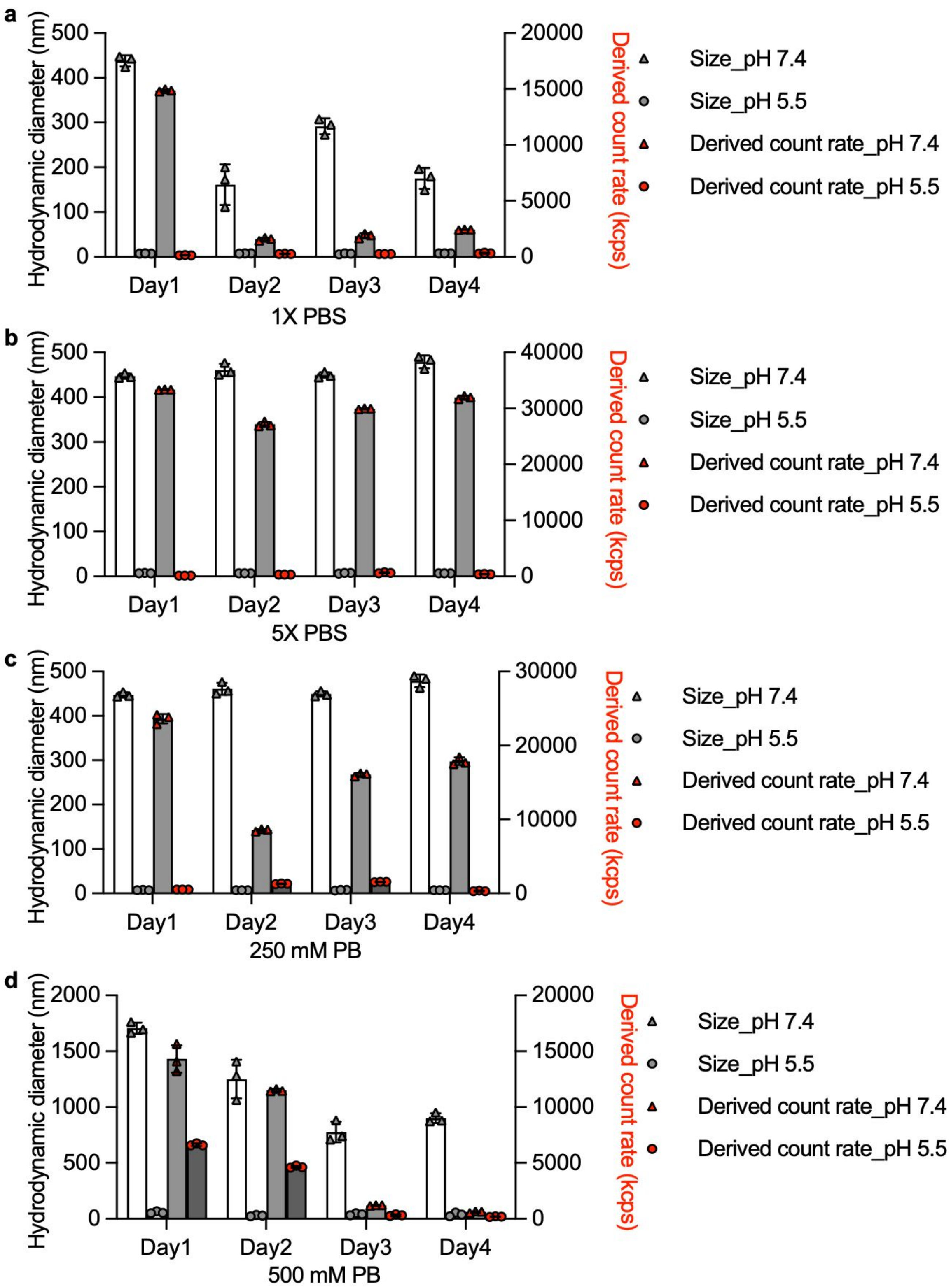

Figure S8. DLS characterization (number-derived size and number-derived count rate) of polymersomes synthesized with 5 mol% DMAEMA when using different purification buffers. a. 1X DPBS **b.** 5X PBS **c.** 250 mM PB. **d.** 500 mM PB were used as purification buffer, respectively (mean values ± standard deviation, n = 3 technical replicates).

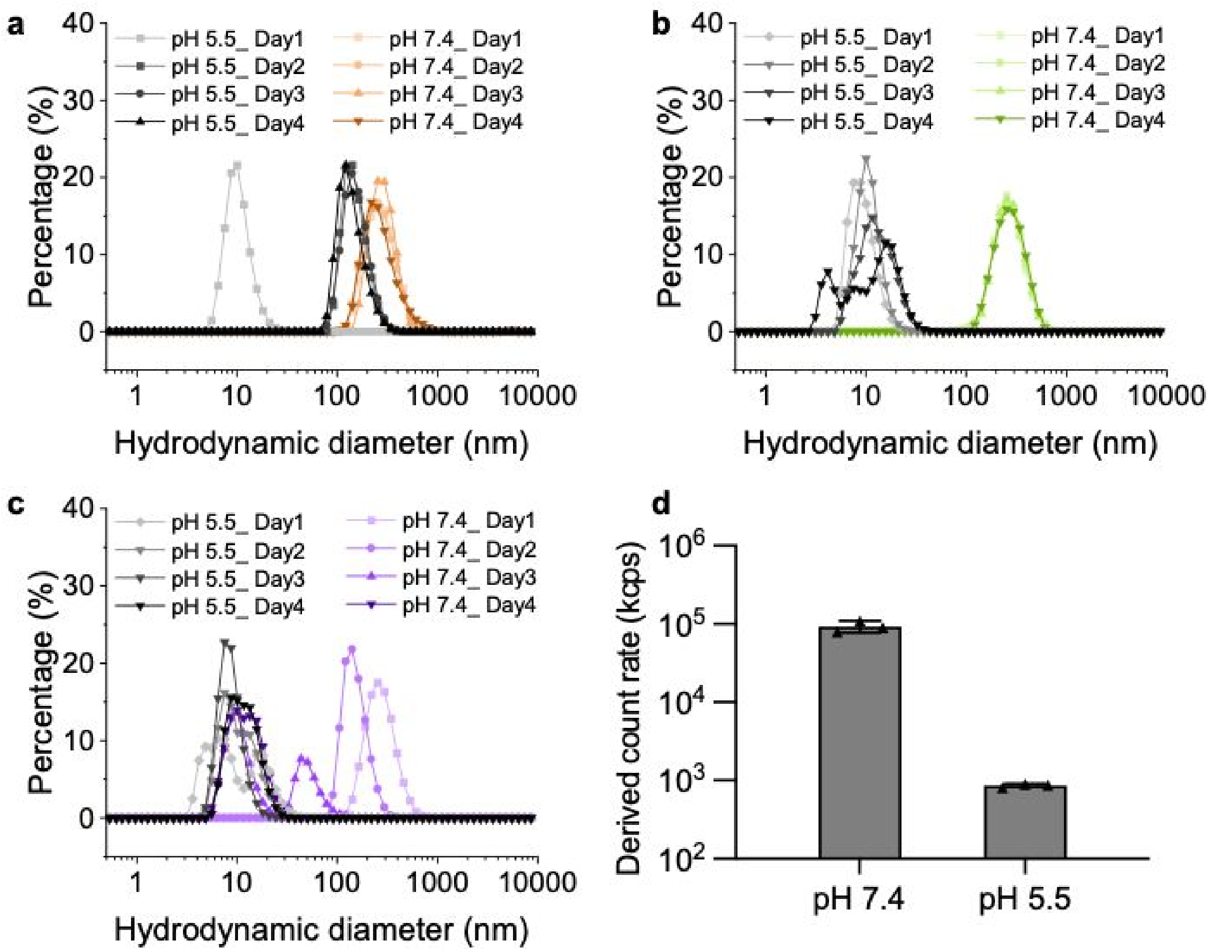

Figure S9. DLS characterization of pH-responsive polymersomes at pH 7.4 and pH 5.5 over 4-day storage. a. Polymersomes with 5 mol% DMAEMA. **b.** Polymersomes with 10 mol% DMAEMA. **c.** Polymersomes with 15 mol% DMAEMA. Sizes are represented at pH 7.4 (intensity size distribution) and pH 5.5 (number size distribution), respectively (mean of n = 3 technical measurements). **d.** Mean derived count rate from (**b**) at day 1, consistent with size measurement in **Figure 3d** (mean value ± standard deviation, n = 3 technical replicates).

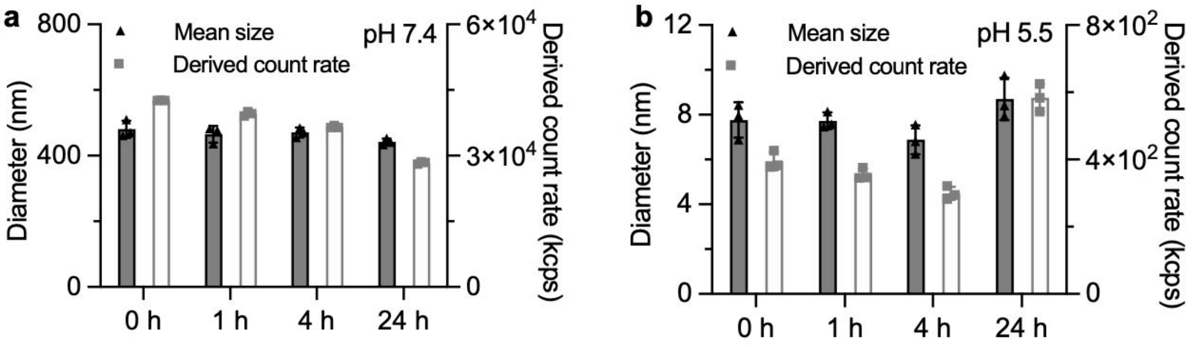

Figure S10. DLS characterization of polymersomes (10 mol% DMAEMA) after incubation at 37°C in DPBS (pH 7.4) and acetate acid buffer (pH 5.5). **a.** Incubation at pH 7.4 (intensity mean) from 0 to 24 hours. **b.** Incubation at pH 5.5 (number mean) from 0 to 24 hours, with same trend for size distribution in **Figure 3 e,f** (mean values ± standard deviation, n = 3 technical replicate).

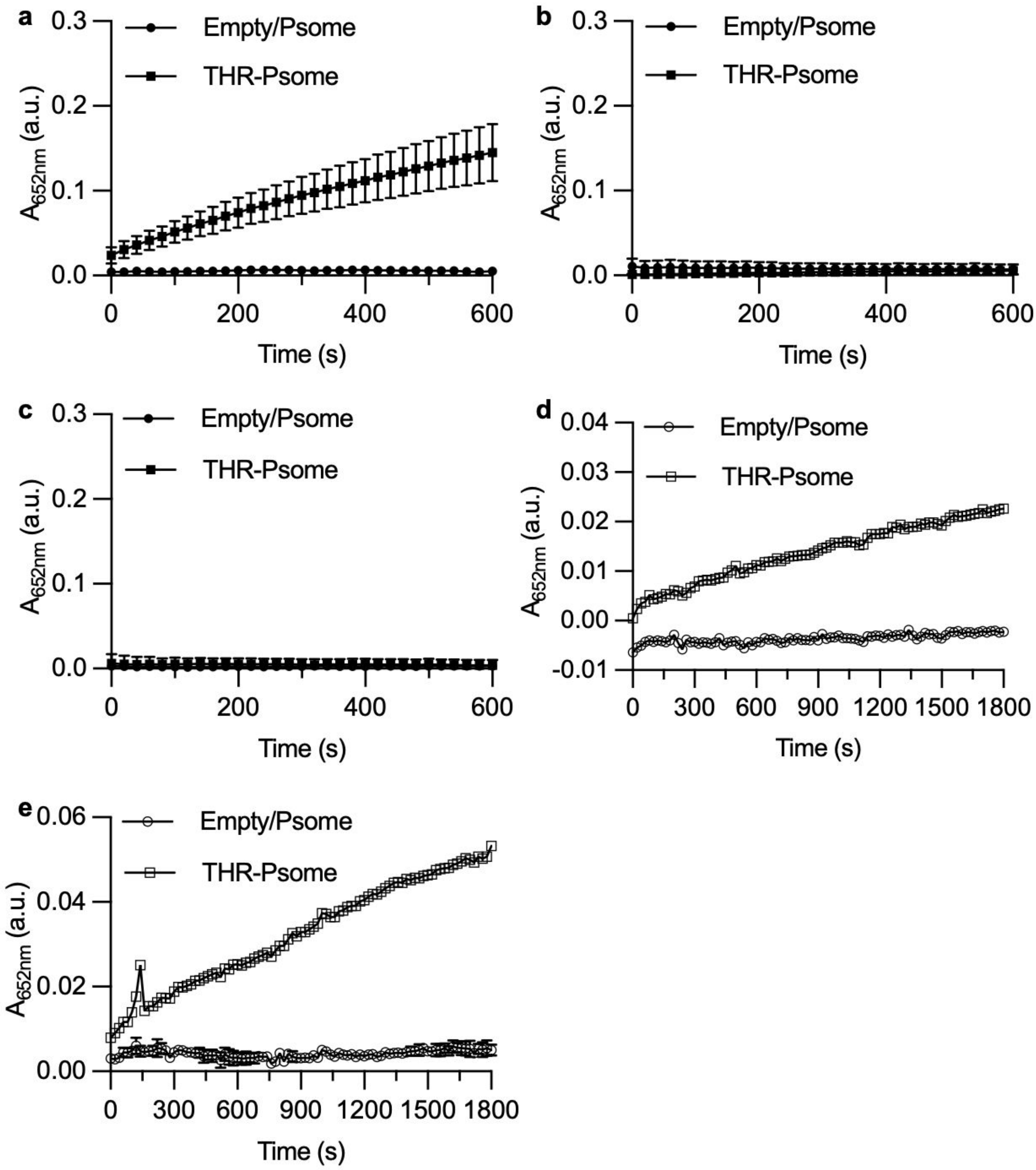

Figure S11. TMB assays on THR-Psome supernatants from purification and purified samples. a-c. Absorbance measurements for TMB assays over time for the (**a**) first, (**b**) second and (**c**) third supernatants from the THR-Psome purification by centrifugation (14,000 × g for 10 min) and resuspension by using 5X PBS. **d, e.** Absorbance measurements for TMB assays on the purified polymersomes encapsulated with and without THR sensor at (**d**) pH 7.4 and (**e**) pH 5.5 (mean values, N = 3 individual replicates, n = 1 technical replicate).

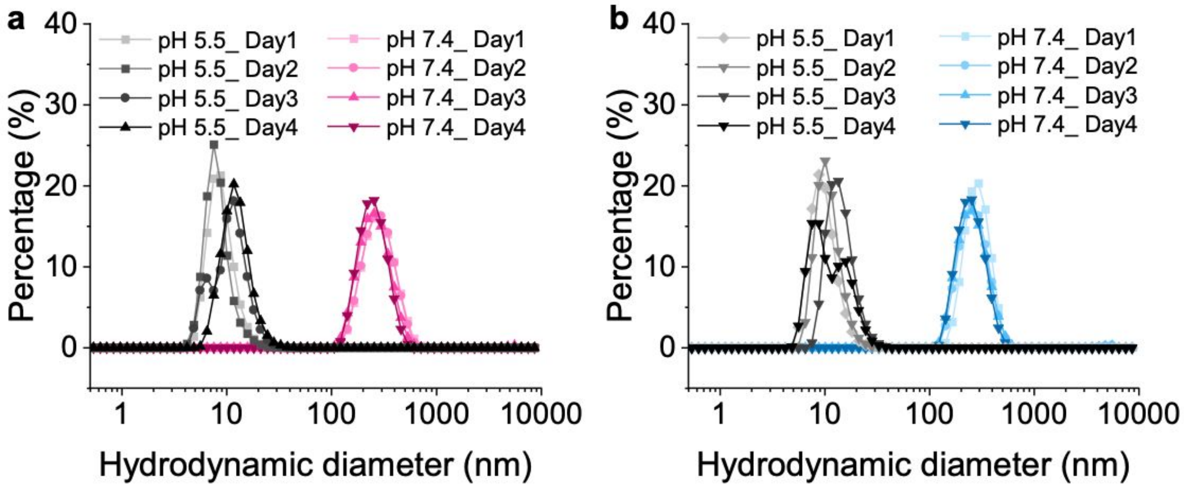

Figure S12. DLS characterization of THR- and MMP-9-Psomes. **a, b**. DLS size distribution of (**a**) THR- and (**b**) MMP-9 Psomes (intensity and number size distribution for measurement at pH 7.4 and 5.5, respectively, mean of n = 3 technical measurements).

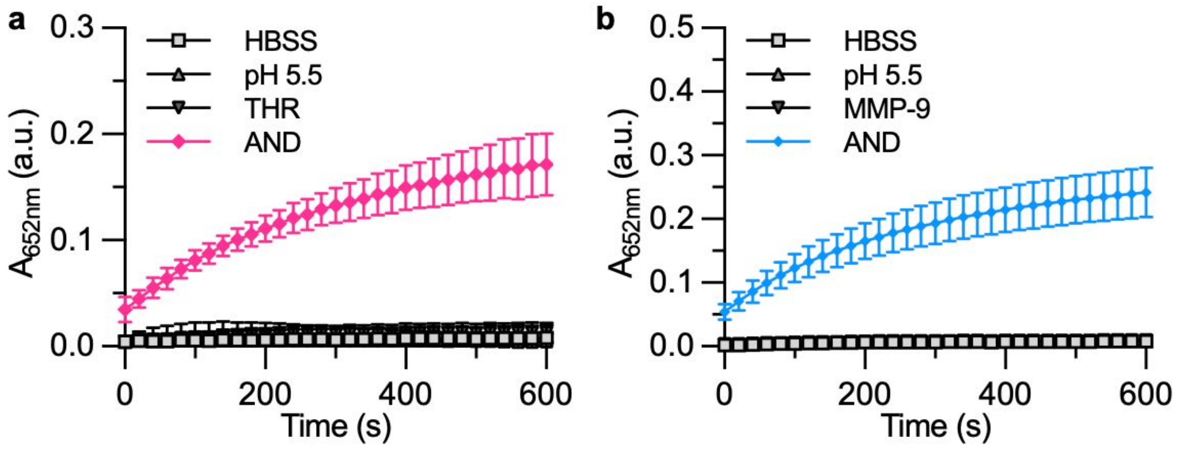

Figure S13. TMB oxidation assays on THR- and MMP-Psomes in different conditions. a. TMB oxidation assay of filtrates from THR-Psomes after incubation in four different treatment conditions. **b.** TMB oxidation assay of filtrates from MMP-Psomes after incubation in four different treatment conditions. Trends are consistent for the calculated onset velocity in Figure 5b, c. Four conditions: (i) pH 7.4 (HBSS buffer), (ii) pH 5.5 (acetate buffer), (iii) enzyme buffer with enzymes (either 50 nM thrombin or MMP-9) at pH 7.4, and (iv) AND gate sensing performed by first incubating in acetate buffer pH 5.5 for 5 min, then pH adjustment back to pH 7.4 by adding bicarbonate buffer pH 9.4 for 5 min, and finally adding 50 nM enzyme in enzyme buffer at pH 7.4 for a further 4-h incubation. Curves are mean of N = 3 biological replicates).

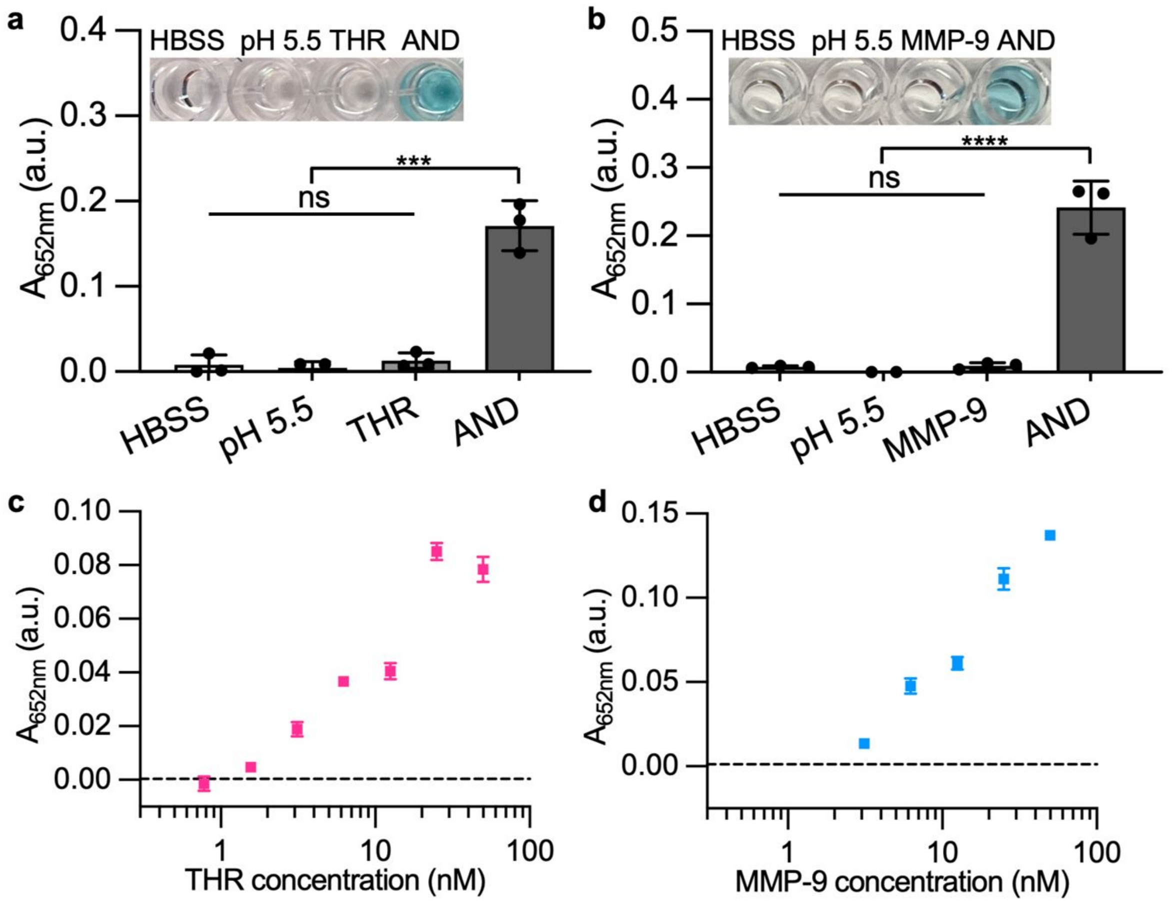

Figure S14. TMB oxidation assays on THR- and MMP-Psomes in different conditions. Endpoint absorbance analysis (A_{652nm} at 10 min) corresponding to **Figure 5b–e.** (**a, b**) TMB assay after incubation of (**a**) THR-Psome and (**b**) MMP-Psome in four different conditions (mean value ± standard deviation, N = 3 biological replicates, n = 3 technical replicates, one-way ANOVA with Tukey's multiple comparisons test, *** p <0.001 for (**a**), **** p <0.0001 for (**b**) and ns: not statistically significant p >0.05). (**c, d**) *In vitro* cleavage performance of (**c**) THR-Psome and (**d**) MMP-Psome in the AND gate sensing condition shown in **Figure 5a**, iv (mean value ± standard deviation, N = 3 biological replicates, n = 3 technical replicates). The dashed line indicates the LoD, derived from the mean background signal plus 3 standard deviations. Trends are consistent for the calculated onset velocity in **Figure 5b-e**. Four conditions: (i) pH 7.4 (HBSS buffer), (ii) pH 5.5 (acetate buffer), (iii) enzyme buffer with enzymes (either 50 nM thrombin or MMP-9) at pH 7.4, and (iv) AND gate sensing performed by first incubating in acetate buffer pH 5.5 for 5 min, then pH adjustment back to pH 7.4 by adding bicarbonate buffer pH 9.4 for 5 min, and finally adding 50 nM enzyme in enzyme buffer at pH 7.4 for a further 4-h incubation.

References

[1] C. N. Loynachan, A. P. Soleimany, J. S. Dudani, Y. Lin, A. Najer, A. Bekdemir, Q. Chen, S. N. Bhatia, M. M. Stevens, *Nat Nanotechnol* **2019**, *14*, 883.
